# Untargeted LC–MS/MS-Based Metabolomic Profiling for the Edible and Medicinal Plant *Salvia miltiorrhiza* Under Different Levels of Cadmium Stress

**DOI:** 10.3389/fpls.2022.889370

**Published:** 2022-07-28

**Authors:** Jun Yuan, Rongpeng Liu, Shasha Sheng, Haihui Fu, Xiaoyun Wang

**Affiliations:** ^1^School of Nursing, Jiangxi University of Chinese Medicine, Nanchang, China; ^2^Research Center for Traditional Chinese Medicine Resources and Ethnic Minority Medicine, Jiangxi University of Chinese Medicine, Nanchang, China; ^3^Key Laboratory of Crop Physiology, Ecology and Genetic Breeding, Ministry of Education, Jiangxi Agricultural University, Nanchang, China

**Keywords:** *Salvia miltiorrhiza*, Cd treatment, LC-MS/MS, discriminating metabolites, metabolic pathways

## Abstract

*Salvia miltiorrhiza*, a medicinal and edible plant, has been extensively applied to treat cardiovascular diseases and chronic hepatitis. Cadmium (Cd) affects the quality of *S. miltiorrhiza*, posing serious threats to human health. To reveal the metabolic mechanisms of *S. miltiorrhiza*'s resistance to Cd stress, metabolite changes in *S. miltiorrhiza* roots treated with 0 (CK), 25 (T1), 50 (T2) and 100 (T3) mg kg^−1^ Cd by liquid chromatography coupled to mass spectrometry (LC–MS/MS) were investigated. A total of 305 metabolites were identified, and most of them were amino acids, organic acids and fatty acids, which contributed to the discrimination of CK from the Cd-treated groups. Among them, *S. miltiorrhiza* mainly upregulated o-tyrosine, chorismate and eudesmic acid in resistance to 25 mg kg^−1^ Cd; DL-tryptophan, L-aspartic acid, L-proline and chorismite in resistance to 50 mg kg^−1^ Cd; and L-proline, L-serine, L-histidine, eudesmic acid, and rosmarinic acid in resistance to 100 mg kg^−1^ Cd. It mainly downregulated unsaturated fatty acids (e.g., oleic acid, linoleic acid) in resistance to 25, 50, and 100 mg kg^−1^ Cd and upregulated saturated fatty acids (especially stearic acid) in resistance to 100 mg kg^−1^ Cd. Biosynthesis of unsaturated fatty acids, isoquinoline alkaloid, betalain, aminoacyl-tRNA, and tyrosine metabolism were the significantly enriched metabolic pathways and the most important pathways involved in the Cd resistance of *S. miltiorrhiza*. These data elucidated the crucial metabolic mechanisms involved in *S. miltiorrhiza* Cd resistance and the crucial metabolites that could be used to improve resistance to Cd stress in medicinal plant breeding.

## Introduction

*Salvia miltiorrhiza* Bge., a well-known plant used as a medicinal and food product, belongs to the Salvia species (Labiatae) and has a wide range of ecological adaptations (Wu, 1977). Its roots contain many metabolites (e.g., phenolic acids, tanshinones, flavonoids, lipids, carbohydrates, carboxylic acids and terpenoids), chiefly phenolic acids, carbohydrates and tanshinones (Tong et al., 2022). Phenolic acids and tanshinones, which are water-soluble active substances and fat-soluble active substances, respectively, are two groups of pharmaceutical components (Commission, 2020). They can promote blood circulation to remove blood stasis, cool blood to remove carbuncles, and clear heart heat to relieve restlessness (Su et al., 2015). Due to their pharmacological actions, they have been widely used in the treatment of various diseases, including coronary heart disease, angina pectoris, tachycardia, and chronic hepatitis (Li et al., 2009; Shi et al., 2019). In addition to tablets, dripping pills, capsules, granules, injections, oral liquids, and sprays, they can be prepared as a vinum, tea or medicined diet (Su et al., 2015; Tan, 2017). Currently, many studies have been conducted on *S. miltiorrhiza*, such as exploring and optimizing its cultivation modes, improving the contents of its active components, and uncovering its pharmacological effects (Shi et al., 2019; Fu et al., 2020; Yan et al., 2020; Lv, 2021). These findings lay a theoretical foundation for the study of ensuring *S. miltiorrhiza* safety based on metabonomics.

Cd, along with arsenic (As), lead (Pb), mercury (Hg) and chromium (Cr), is a toxic nonessential metal and it accumulates in organisms with a long half-life of approximately 25-30 years (Kabata and Pendias, 1992; Genchi et al., 2020b). Over the past century, various human activities have resulted in environmental Cd pollution (Rahimzadeh et al., 2017; Genchi et al., 2020a,b). Characterized by strong bioaccumulation, high bioavailability, and strong biotoxicity, Cd can cause the wilting of leaves, the browning of roots, and even the death of plant cells, leading to a decline in plant yield and quality (Sarangthem et al., 2011; Wang et al., 2019; Grajek et al., 2020). Cd accumulates in the human body through the food chain and causes irreversible damage (Valverde et al., 2001; Satarug et al., 2003; Filipič, 2012). Therefore, plant Cd contamination has attracted much attention from researchers.

With the aggravation of heavy metal pollution in soil, heavy metals (e.g., Cd, As, Pb, Hg) have become important pollutants in traditional Chinese medicine (TCM), which would restrain the sustainable development of the TCM industry (Meng et al., 2009; Yan et al., 2012). According to previous studies, Cd stress could inhibit growth, accumulate Cd residue and affect secondary metabolites of *S. miltiorrhiza* roots (Zhang et al., 2013; Wei et al., 2020). However, little has been published on the Cd-resistance mechanisms of *S. miltiorrhiza* based on metabolomics.

In the present study, taking *S. miltiorrhiza* seedlings under different levels of Cd treatment as the research object, metabolites of *S. miltiorrhiza* roots were determined by LC–MS/MS in a pot experiment. The objective of this study was to investigate the main metabolites of *S. miltiorrhiza* in resistance to Cd under different levels of Cd stress and how *S. miltiorrhiza* resists Cd stress based on the metabolome. This study provides deep knowledge of the response to Cd stress in *S. miltiorrhiza* and lays a foundation for further revealing the Cd resistance mechanisms of *S. miltiorrhiza*, which can be used as a reference by plant breeders and forest managers.

## Materials and Methods

### Plant Materials

The *S. miltiorrhiza* seedlings used in the study were purchased from the plantation of *S. miltiorrhiza* in Pingyi County, Shandong Province (35°30' N, 117°35' E). The area has a temperate seasonal climate with an average elevation of 87.9 m. The annual mean precipitation, air temperature, and average relative humidity are 836.0 mm, 14.3 °C and 67.1%, respectively (Lu, 2020; Tian et al., 2021). Healthy seedlings with approximately 4 basal leaves were cultivated from the upper and middle parts of the annual root. They were identified as *S. miltiorrhiza* Bge. By Associate professor Xiaoyun Wang from the Research Center for Traditional Chinese Medicine Resources and Ethnic Minority Medicine of Jiangxi University of Chinese Medicine (JXUCM).

Healthy and disease-free seedlings were selected and planted in Shennong garden of JXUCM. The physical and chemical properties of the topsoil (0–20 cm) in the planting area of *S. miltiorrhiza* in Shennong garden were as follows: total nitrogen 0.30 g kg^−1^, total phosphorus 0.28 g kg^−1^, total potassium 27.15 g kg^−1^, available nitrogen 0.01 g kg^−1^, available phosphorus 0.01 g kg^−1^, available potassium 0.08 g kg^−1^, organic matter 1.97 g kg^−1^, pH 4.6, and total Cd 0.92 mg kg^−1^. The total Cd content was lower than the critical value of Cd (1.0 mg kg^−1^) in soils, which could ensure the normal growth of plants.

The indicated amounts of cadmium chloride hemi (pentahydrate) (CdCl_2_·2.5H_2_O) were mixed well with sieved topsoils from Shennong garden. Based on the study of Zhang et al. (2013) and Wei et al. (2020), four Cd treatment levels were set in the present study as follows: 0 (CK), 25 mg kg^−1^ (T1), 50 mg kg^−1^ (T2), and 100 mg kg^−1^ (T3). Subsequently, 2 kg of the soils were placed a flower pot (16 × 17 cm) and incubated for 30 d. Then, the *S. miltiorrhiza* seedlings, which had grown in Shennong garden for 30 d, were transplanted into the pots, and each pot included one seedling. There were three repetitions at each level and three seedlings in each repetition. After 15 d of treatment, root samples were collected, washed with ultrapure water, quickly frozen with liquid nitrogen, and transferred to −80°C until further metabolomic analysis. Voucher specimens (No. DS-001) were deposited in a public herbarium in the Research Center for Traditional Chinese Medicine Resources and Ethnic Minority Medicine of JXUCM.

### Metabolite Extraction

A 25 mg fresh sample in a 500 μL mixture of methanol and water (3:1, v/v) (including an isotope-labeled internal standard mixture) was ground at 35 Hz for 4 min and lysed in an ultrasonic water bath for 5 min. After sitting at −40°C for 1 h, the samples were centrifuged at 12000 rpm for 15 min at 4°C. The supernatant was stored at −80°C until liquid chromatography-tandem mass spectrometry (LC–MS/MS) analysis. Quality control (QC) samples were prepared with a mix of the supernatants from all samples.

### LC–MS/MS Analysis

Ultrahigh-performance liquid chromatography and quadrupole orbital well hybrid TM mass spectrometry coupled with an Acquity UPLC HSS T3 liquid chromatographic column were conducted to separate the target compounds.

The chromatographic conditions were as follows: an Acquity UPLC HSS T3 liquid chromatographic column (2.1 mm × 100 mm, 1.8 μm) was used; the column temperature was 35°C; mobile phase A and phase B were the aqueous phase (containing 5 mmol L^−1^ ammonium acetate and 5 mmol L^−1^ acetic acid) and acetonitrile, respectively; gradient elution was carried out (0–0.7 min, 1% B; 0.7–9.5 min, 1–99% B; 9.5–11.8 min, 99% B; 11.8–12.0 min, 99–1% B; 12–14.8 min, 1% B); the flow rate was 0.5 mL min^−1^ and the injection volume was 3 μL.

A Thermo Q Exactive HFX mass spectrometer was used to collect the primary and secondary mass spectrometry data of the samples under the control of Xcalibur 4.0.27 (Thermo Scientific). The detailed parameters are presented in Supplementary Table S1.

### Metabolite Data Acquisition

The raw data were converted into mzXML format with the software ProteoWizard (https://proteowizard.sourceforge.io/), and the recognition, extraction, alignment and integration of the peaks was conducted with the R package (XCMS as the core). Then, the MS2 database was applied for metabolite annotation. The cutoff for annotation was set at 0.3. Data with no definite substance name and no spectral ratio or substances with a missing quantity greater than 50% in the comparison group samples were filtered and removed. For substances with a missing quantity less than 50%, the K-nearest neighbor (KNN) algorithm was used to simulate the missing value. Finally, the total ion current (TIC) or internal standard (IS) of each sample was used to normalize the data, and 305 metabolites were identified for further analysis.

### Data Analysis

Unsupervised principal component analysis (PCA) was performed to analyze the distribution of root samples in CK, T1, T2 and T3. Supervised orthogonal partial least squares-discriminant analysis (OPLS-DA) was used to distinguish the metabolic profiles of the roots between the CK and each Cd-added group (T1, T2 and T3). To test the OPLS-DA model, a cross-validation residual variance test (CV-ANOVA test) and 200 permutation tests were carried out. The model with *p* < 0.05 in the CV-ANOVA test or R2 > 0.7 and Q2 > 0.4 in the permutation test was reliable (Yuan et al., 2020). The variable importance in projection (VIP) was vital for explaining the data of the OPLS-DA model. The discriminating metabolites were defined based on a VIP value above 1 and a *p* value below 0.05 (Yuan et al., 2020). Metabolic pathway analysis of these discriminating metabolites was conducted. Meanwhile, one-way ANOVA was performed to calculate the variability in the relative contents of the metabolites.

Multivariate statistical analysis (PCA and OPLS-DA) and validation of the OPLS-DA model were carried out with SIMCA-P 14.1 (Umetrics, Sweden). Univariate statistical analysis and one-way ANOVA were conducted with IBM SPSS Statistics 20.0 (SPSS, Inc., IBM Corp, New York, USA). The metabolic pathway analysis was carried out with the online software MetaboAnalyst 4.0 (http://www.Metaboanalyst.ca/faces/ModuleView.xhtml). The heatmap was drawn by online software (https://matrix2png.msl.ubc.ca/bin/matrix2png.cgi). The Venn diagrams were drawn by Venny 2.1 (https://bioinfogp.cnb.csic.es/tools/venny/index.html).

## Results

### Metabolites Responding to Different Levels of Cd Stress

By observing the differences in the peak height of the internal standard between the QC samples and the peak conditions of the internal standard in the blank samples, this study determined whether the instrument was stable and whether there were residues in the detection process. Supplementary Figure S1 shows that both the retention time and the response strength of the internal standard in the QC samples were stable, and the data acquisition stability of the instrument was excellent. Supplementary Figure S2 shows that no obvious peaks other than the internal standards were detected in the blank samples.

**Figure 1:**
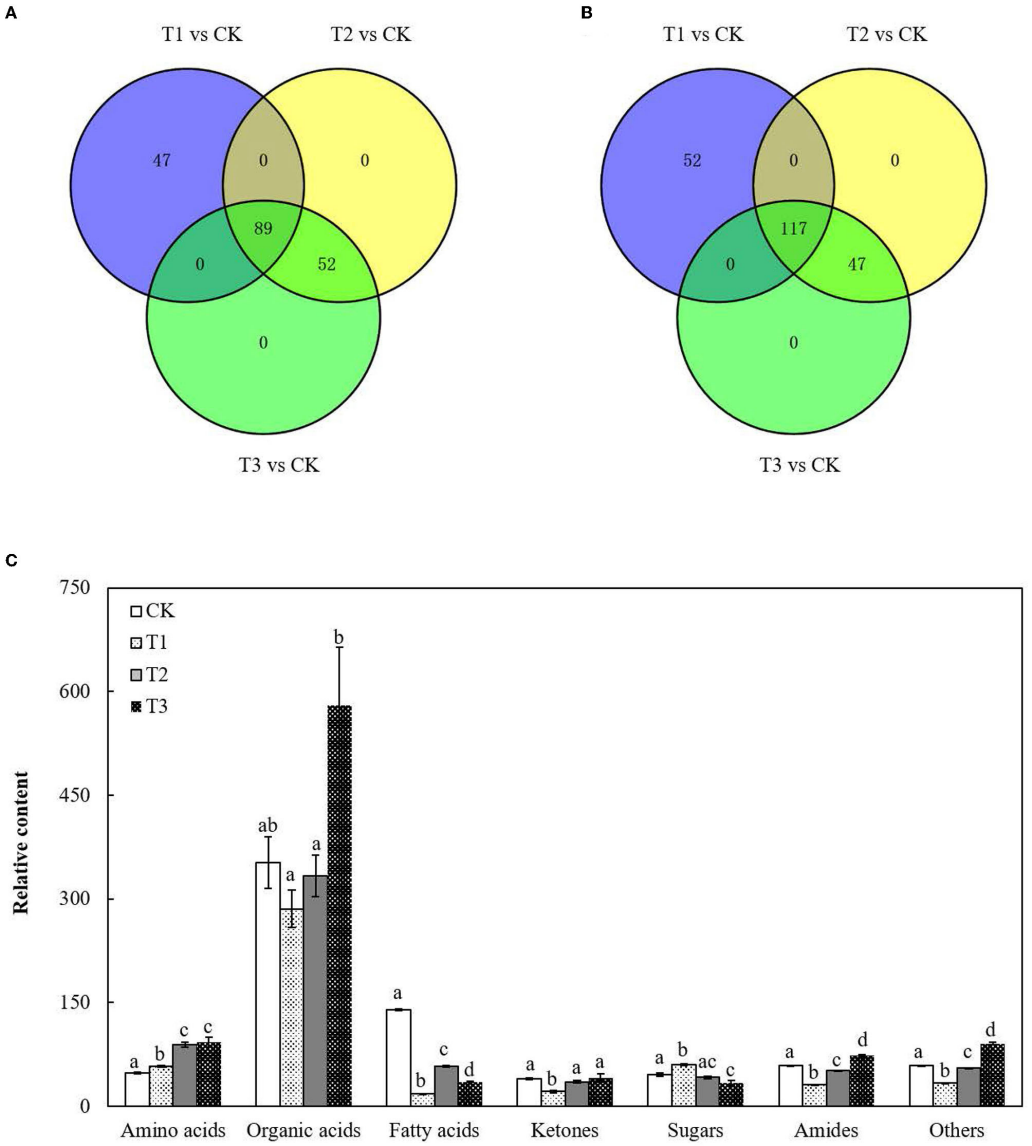
Variation in the total metabolites in *S. miltiorrhiza* roots under different levels of Cd stress. **(A,B)** represent changes in the total number of upregulated and downregulated metabolites in *S. miltiorrhiza* roots with different levels of Cd treatment, respectively; different colors in **(A,B)** represent different sources of upregulated metabolites and downregulated metabolites, respectively; **(C)**, Total relative amino acid, organic acid, fatty acid, ketones, sugar, amide and others content in *S. miltiorrhiza* roots; Different lowercase letters on the columns of the same metabolite indicate significant differences within each treatment in the roots (*p* < 0.05); vertical bars above the columns indicate the standard error of each mean (mean ± SE).

The metabolite profiles of the roots were investigated using an untargeted global metabolomic platform with LC–MS/MS. A total of 305 metabolites were annotated and quantified in *S. miltiorrhiza* roots with different levels of Cd treatment (Supplementary Table S2), which could be categorized into seven major groups based on their molecular structure: amino acids, organic acids, fatty acids, ketones, sugars, amides, and others (Figure 1C; Supplementary Table S3).

There were 136 upregulated metabolites and 169 downregulated metabolites in T1 and 141 upregulated metabolites and 164 downregulated metabolites in both T2 and T3 (Figures 1A,B). Proportions of the metabolites in the roots changed in response to Cd addition (Figure 1C; Supplementary Table S3). Compared to CK, the total content of amino acids increased by 0.2-fold in T1, approximately 0.9-fold in T2 and T3 (*p* < 0.05), and the total content of fatty acids decreased by 87% in T1, 59% in T2 and 75% in T3 (*p* < 0.05). There were no significant differences in the total content of organic acids between CK and each Cd-added group (Figure 1; Supplementary Table S3).

### Discriminating Metabolites in Roots Between CK and Each Cd-Added Group

**Figure 2:**
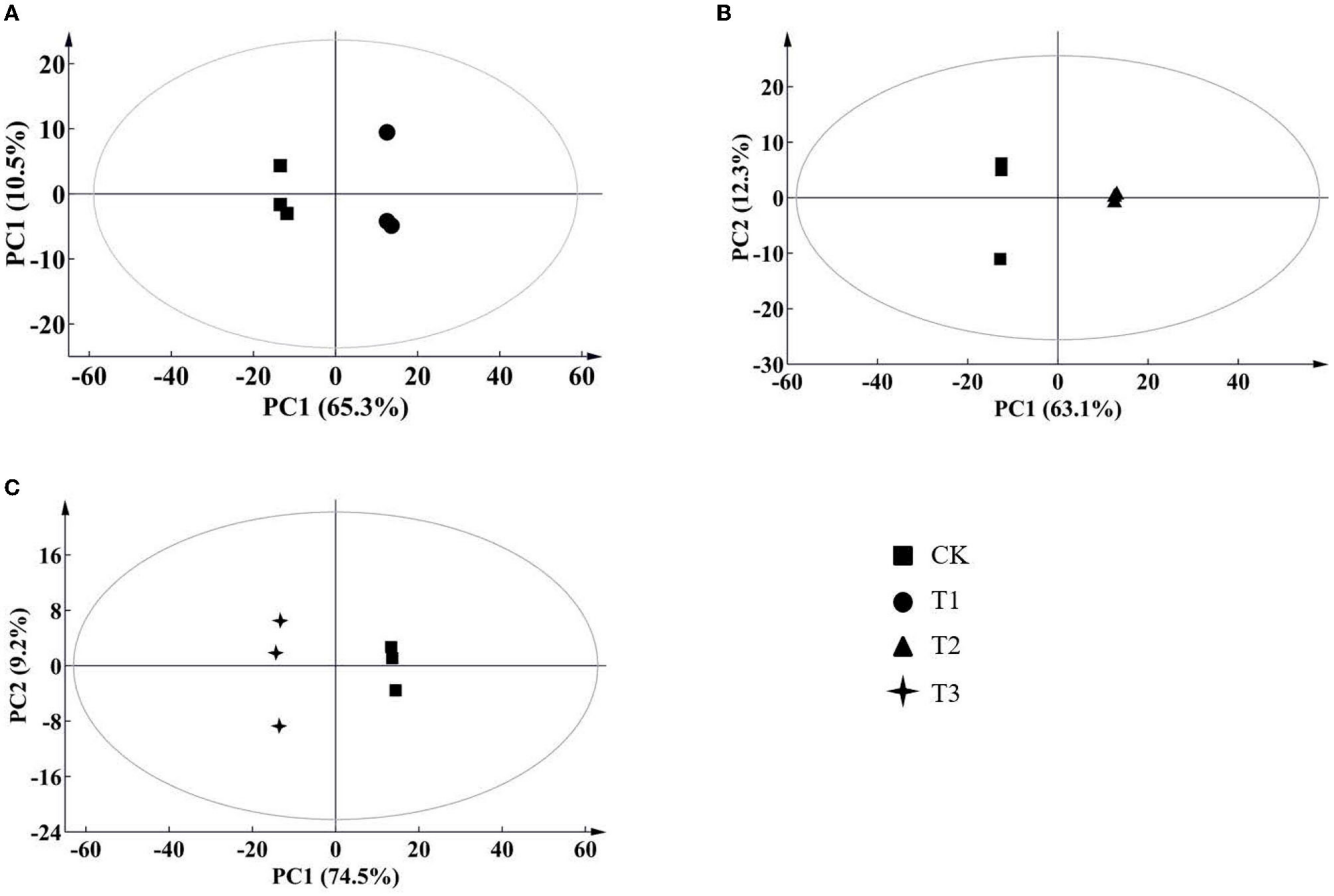
Score plots of principal component analysis (PCA) for metabolomic data from *S. miltiorrhiza* roots under different levels of Cd stress. PC1, the first principal component; PC2, the second principal component. The ellipse indicates Hotelling's T2 (95%); **(A–C)** stand for score plots PCA of metabolite data obtained from LC–MS/MS in *S. miltiorrhiza* roots of T1 (25 mg/kg Cd group) and CK (control group), of T2 (50 mg/kg Cd group) and CK, and of T3 (100 mg/kg Cd group) and CK, respectively.

**Figure 3:**
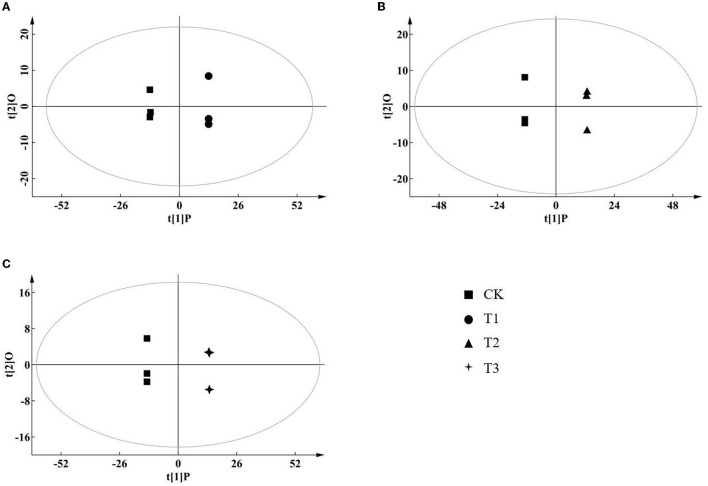
Score plots of orthogonal projections to latent structures discriminant analysis (OPLS-DA) of metabolite data in *S. miltiorrhiza* roots. **(A–C)** stand for score plots OPLS-DA of metabolite data obtained from LC–MS/MS in *S. miltiorrhiza* roots of T1 (25 mg/kg Cd group) and CK (control group), of T2 (50 mg/kg Cd group) and CK, and of T3 (100 mg/kg Cd group) and CK, respectively.

**Figure 4:**
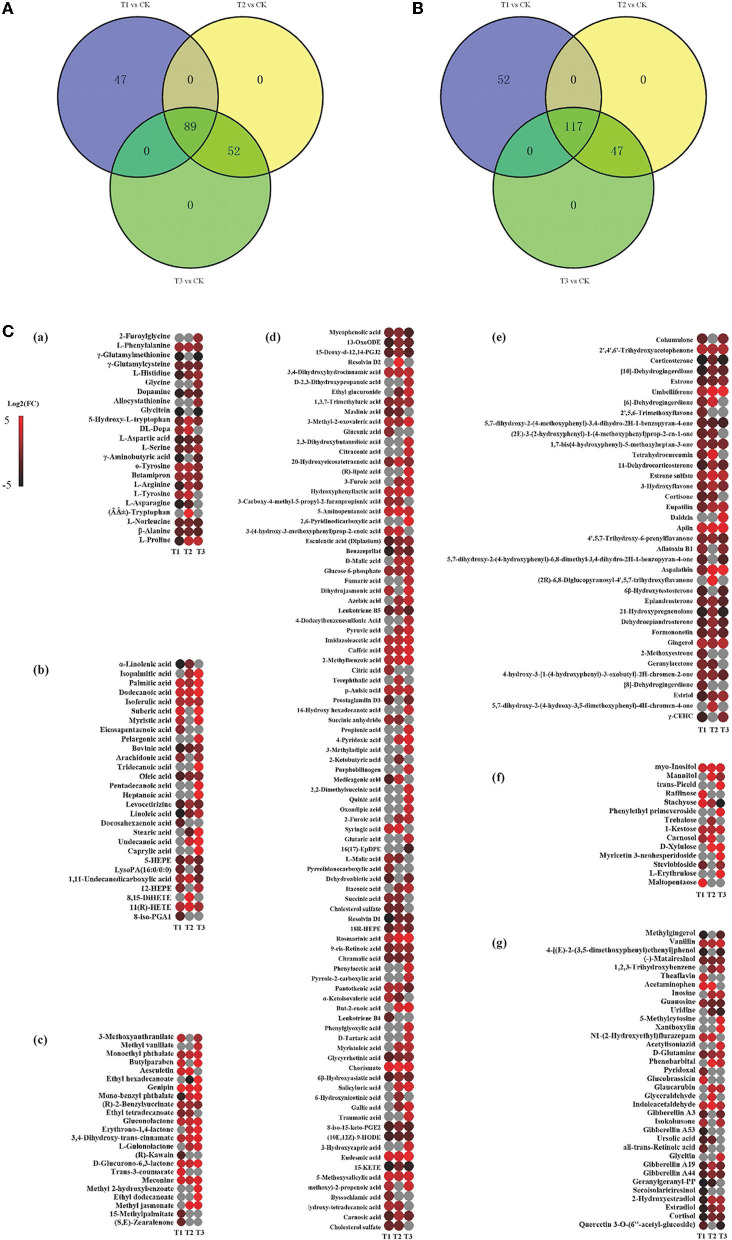
Variation in the discriminating metabolites in *S. miltiorrhiza* roots under different levels of Cd stress. **(A,B)** stand for overlap of the upregulated and downregulated discriminating metabolites of *S. miltiorrhiza* roots in response to different Cd treatments, respectively; different colors in **(A,B)** represent different sources of upregulated discriminating metabolites, and downregulated discriminating metabolites, respectively; **(C)**, Heatmap analysis of amino acids ((Li et al.), fatty acids (b), esters (c), organic acids (d), ketones (e), sugars (f) and others (g) of the discriminating metabolites in *S. miltiorrhiza* roots between the control and Cd stressed groups; T1, T2 and T3 stand for discriminating metabolites in roots between the control and 25 mg kg^−1^ Cd group (T1), 50 mg kg^−1^ group (T2), and 100 mg kg^−1^ Cd group (T3), respectively; Log2(FC), an estimate of the log2-transformed ratio of the relative content of metabolites in *S. miltiorrhiza* roots of the Cd treated group to that of the control group. The colors indicate the log2 transform of the ratios for the relative content of metabolites between the control and Cd treated groups in *S. miltiorrhiza* roots, ranging from black (low level) to red (high level). The gray ellipses stand for not discriminating metabolites.

According to the results of the PCA, root samples from CK could be separated from the Cd-treated groups (Figure 2). Based on the reliable OPLS-DA model with a *p* value below 0.05 (Figure 3; Supplementary Figure S3A), *S. miltiorrhiza* roots in T1 and CK could be distinguished by metabolites with major contributions from amino acids, organic acids, and fatty acids (Figure 4): 174 discriminating metabolites were selected, 56 of which were upregulated and 118 of which were downregulated. Among them, most fatty acids (especially α-linolenic acid, linoleic acid (C18:2), and oleic acid (C18:1)) and organic acids were downregulated, with chorismate and o-tyrosine contributing the most to the upregulation of organic acids and amino acids in *S. miltiorrhiza* roots, respectively.

Similarly, based on the significant OPLS-DA model (Supplementary Figures S3B,C), the root samples could be significantly distinguished by metabolites with the major contributions from 161 metabolites (81 of them were upregulated and 80 were downregulated) in T2 and CK and from 191 metabolites (120 of them were upregulated and 71 were downregulated) in T3 and CK. Major contributors to the differences were amino acids, organic acids, and fatty acids (Figure 4): compared to CK, in T2, all of these amino acids (especially DL-tryptophan, L-aspartic acid, and L-proline) were upregulated, and most fatty acids (e.g., linoleic acid, oleic acid) were downregulated, with chorismate contributing the most to the upregulation of the organic acids (Figure 4); in T3, most of the amino acids (especially L-proline, L-serine and L-histidine), organic acids (especially eudesmic acid and rosmarinic acid) and all of the saturated fatty acids (e.g., stearic acid (C18:0)) were upregulated, and oleic acid was downregulated (Figure 4).

### Metabolic Pathways Involving All Discriminating Metabolites

**Figure 5:**
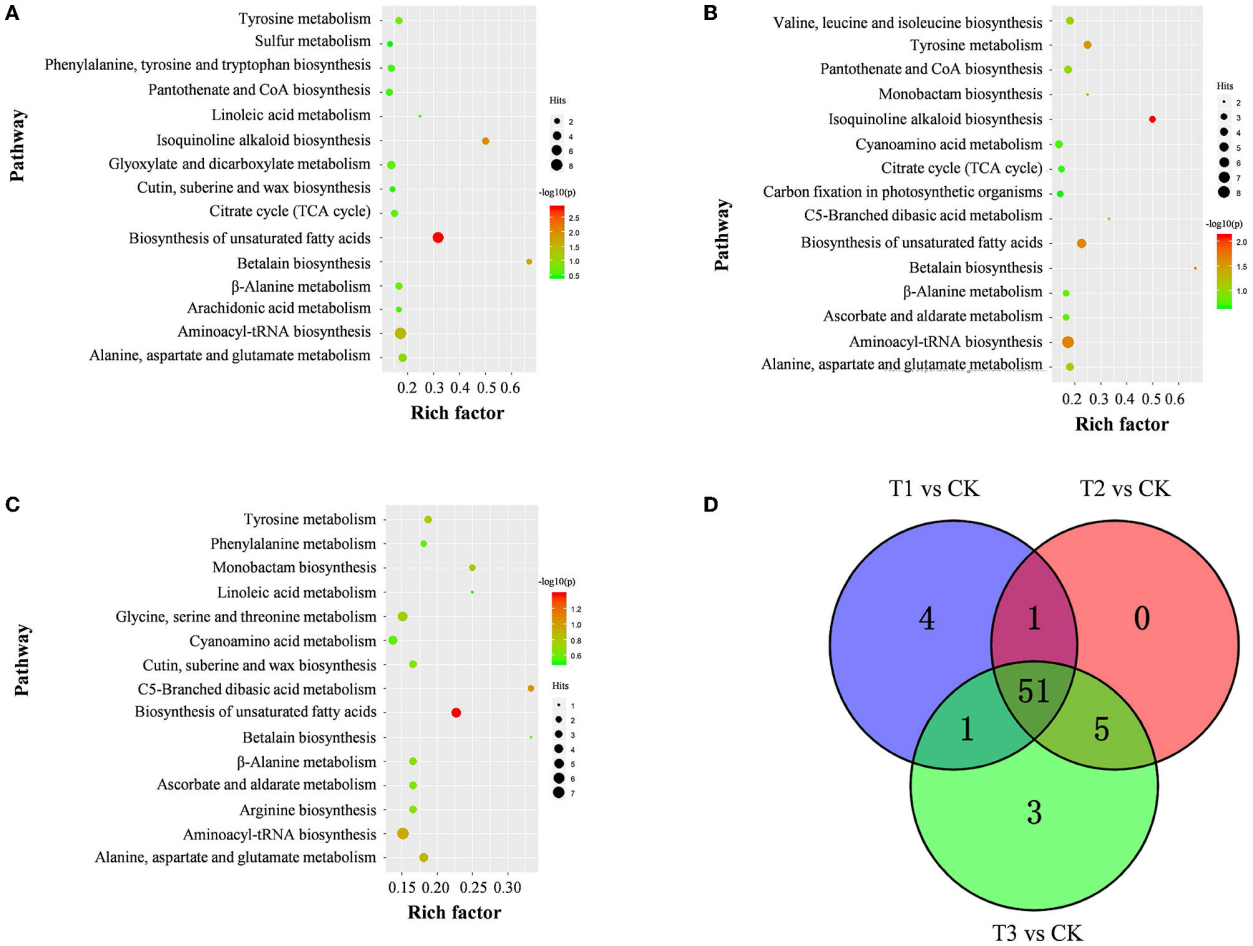
Pathway of discriminating metabolites in *S. miltiorrhiza* roots between the control and Cd-stressed groups. **(A–C)** represent pathways of discriminating metabolites between CK and T1 **(A)**, T2 **(B)**, and T3 **(C)**, respectively; the size and color of the bubble represent the number hits and -log10(p) values for each pathway from pathway analysis; **(D)**, overlap of metabolic pathways discriminating metabolites of *S. miltiorrhiza* roots based on KEGG in response to different levels of Cd stress; different colors represent different sources of metabolic pathways for discriminating metabolites.

**Figure 6:**
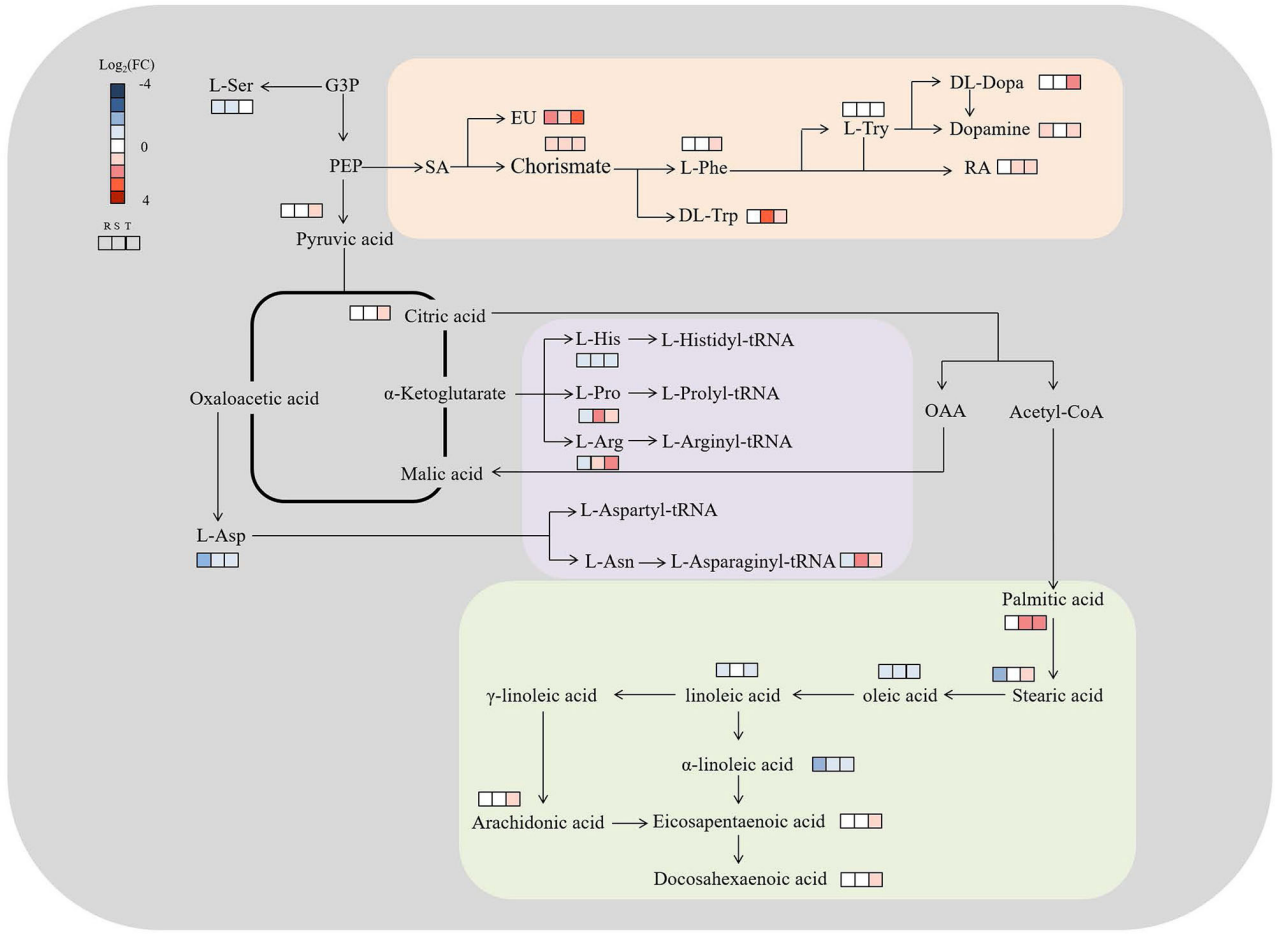
Metabolic map of *S. miltiorrhiza* roots based on LC–MS/MS with different levels of Cd stress. Metabolisms in orange were involved in isoquinoline alkaloid biosynthesis, betalain biosynthesis, and tyrosine metabolism, and purple and green were involved in aminoacyl-tRNA biosynthesis and unsaturated fatty acids biosynthesis, respectively; G3P, PEP, L-Ser, SA, EU, L-Phe, L-Try, RA, L-Asp, L-His, L-Pro, L-Arg and L-Asn represent glucose-3-phosphate, phosphoenolpyruvic acid, L-serine, shikimic acid, eudesmic acid, L-phenylalanine, L-tyrosine, rosmarinic acid, L-aspartic acid, L-histidine, L-proline, L-arginine, and L-asparagine, respectively; Log2(FC), an estimate of the log2-transformed ratio of the relative content of metabolites in *S. miltiorrhiza* roots of the Cd treated group to that of the control group.

The discriminating metabolites were annotated into the Kyoto Encyclopedia of Genes and Genomes (KEGG) database, and 65 metabolic pathways were obtained (Figure 5A; Supplementary Table S4). Among them, biosynthesis of unsaturated fatty acids, isoquinoline alkaloid biosynthesis, betalain biosynthesis, aminoacyl-tRNA biosynthesis, and tyrosine metabolism were the significantly enriched metabolic pathways (*p* < 0.05) (Figure 5B; Supplementary Table S4). In addition, a metabolic map of the resistance process was developed based on these results (Figure 6).

## Discussion

To the best of our knowledge, this study is the first to illustrate how the medicinal and food plant *S. miltiorrhiza* resists abiotic stress in terms of the metabolome. The discussions focused on changes in the metabolic profiles of roots and the identification of metabolites that played key roles in metabolic regulation under different levels of Cd stress.

### Global Responses of the Metabolome Under Cd Stress

Our results clearly revealed that amino acids, organic acids and fatty acids in *S. miltiorrhiza* roots played essential roles in resisting Cd stress. As the basic components of proteins, amino acids can participate in the regulation of ion transport and nitrogen metabolism and play vital roles in resisting abiotic stress in plants (Sharma and Dietz, 2006; Xu et al., 2012; Tian, 2021). Organic acids are a class of compounds containing carboxyl groups (excluding amino acids), most of which can combine with metal ions or alkaloids (Kuang, 2017). Fatty acids and their derivatives are essential energy stores and the main components of membrane lipids (Chaffai et al., 2009; Liu et al., 2014; Li-Beisson et al., 2016). Due to the important roles of these three classes of metabolites in plant survival and stress resistance (Liu et al., 2014; Panchal et al., 2021; Trovato et al., 2021), in this study, the changes in the proportions of amino acids, organic acids, and fatty acids were larger than those of the other metabolites in *S. miltiorrhiza* roots under Cd treatment (Figure 1).

In comparison with CK, in T1, T2 and T3, the total relative contents of the amino acids significantly increased (*p* < 0.05), the total relative contents of the fatty acids significantly decreased (*p* < 0.05), and the total relative contents of the organic acids presented no significant differences (Figure 1; Supplementary Table S3). In disagreement with these results, in *Oryza sativa* L. roots, Cd at a low content could promote the production of amino acids and organic acids, and Cd at a high content could inhibit the production of amino acids and organic acids (Tang et al., 2016), while in agreement with our results, there was a significant decline in the production of fatty acids in *Sedum plumbizincicola* L. roots under Cd stress (Sun et al., 2020). This might result from the fact that the plant metabolic response to abiotic stress is affected by the stress modes, stress intensity, and plant species (Hu and Xu, 2014; Zhang and Chen, 2021).

### Amino Acids Play Vital Roles in Resisting Cd Stress

As reported above, amino acids were the main contributors to the differences in *S. miltiorrhiza* roots under different levels of Cd stress (Figure 4). Well known as an abiotic and biotic stress indicator, proline can function as a hydroxyl radical scavenger and it plays a vital role in the adjustment to osmotic stresses in plants (Sharma and Dietz, 2006; Zemanov et al., 2017). Tyrosine is the precursor of many metabolites (including tocopherol, plastoquinone and ubiquinone) that are essential to the survival and stress resistance of plants (Kilgore and Kutchan, 2016; Cassels and Sáez-Briones, 2018; Xu et al., 2020). Tryptophan, an aromatic amino acid, plays important roles in the regulation of plant development and it acts as a precursor for the biosynthesis of the hormone auxin (Sanjaya et al., 2008; Liu et al., 2011a). Histidine plays important roles in the regulation of the biosynthesis of other amino acids and in the chelation and transport of metal ions (Stepansky and Leustek, 2006). Aspartic acid can be fed into the synthesis of other amino acids (e.g., lysine, methionine and threonine) (Angelovici et al., 2009). Serine is essential for the synthesis of proteins and other biomolecules, including nucleotides and serine-derived lipids (e.g., phosphatidylserine and sphingolipids), and is involved in the resistance to various stresses (Ho and Saito, 2001; Waditee et al., 2007; Ros et al., 2013). Due to the different roles of these amino acids in the stress resistance of plants, with different levels of Cd treatment, the levels of amino acids playing vital roles in resisting Cd stress were different (Benral and McGrath, 1994; Wang et al., 2015; Cosio and Renault, 2020) (Figures 5, 6).

In the present study, o-tyrosine contributed more to the upregulation of the discriminating amino acids between T1 and CK; DL-tryptophan, L-aspartic acid, and L-proline contributed more to the upregulation of the discriminating amino acids between T2 and CK, and L-proline, L-serine and L-histidine contributed more to the upregulation of the discriminating amino acids between T3 and CK (Figure 4; Supplementary Table S2). This illustrated that there were different Cd stress resistance patterns of amino acids in *S. miltiorrhiza* roots under different levels of Cd stress. Under moderate Cd (T2) stress, *S. miltiorrhiza* roots might mainly increase the relative contents of DL-tryptophan, L-aspartic acid, and L-proline to resist Cd, but under high-level Cd (T3) stress, the roots might mainly increase the relative contents of L-proline, L-serine and L-histidine to resist Cd. Inconsistent with this, compared to CK, the proline content declined significantly and the aspartic acid content increased significantly with moderate Cd treatment; and the histidine content increased significantly and the serine content declined significantly with high Cd treatment (*p* < 0.05) of *Crassocephalum crepidioides* (Zhu et al., 2018). As the level of Cd increased, the contents of tryptophan and proline increased significantly, and the L-aspartic acid content declined significantly in *Solanum nigrum* (Xu et al., 2012). These differences might be caused by the differences in the Cd sensitivity of different plants or by the different methods of applying Cd stress (Zhang and Chen, 2021).

### Organic Acids Play Vital Roles in Resisting Cd Stress

Our study revealed that based on the changes in the relative contents of organic acids in *S. miltiorrhiza* roots with different levels of Cd treatment, chorismate and eudesmic acid in T1, chorismate in T2, and eudesmic acid and rosmarinic acid in T3 were upregulated. In the present study, organic acids were among the main contributors in distinguishing CK samples from Cd treated samples (T1, T2 and T3) (Figure 4; Supplementary Table S2): most organic acids showed a decreasing accumulation, with chorismate and eudesmic acid contributing the most to the upregulation of organic acids in T1; most organic acids showed an increasing accumulation, with chorismate contributing most to the upregulation of organic acids in T2; and most organic acids presented an increasing accumulation (especially eudesmic acid and rosmarinic acid) in T3.

Chorismate could function as a key branching point between primary and secondary metabolisms, as well as being a precursor of aromatic amino acids (e.g., tryptophan, phenylalanine, tyrosine) and hormonal substances (e.g., indoleacetic acid and salicylic acid) that play essential roles in plant metabolism (Kristin and Michael, 2010). Eudesmic acid and rosmarinic acid are polyphenol derivatives and phenol compounds, respectively, and both have strong antioxidation activity and strong hydroxyl radical scavenging activity (Lv et al., 2005; Wang et al., 2014; Korkmaz et al., 2018). Under Cd stress, the elevation of intracellular chorismite would result in some pathways, including the biosynthesis of aromatic amino acids (e.g., phenylalanine and tryptophan) and secondary metabolites (e.g., polyphenols and flavonoids) (Figure 6; Supplementary Table S2) (Malik, 1979), and the activity of the enzymes involved in phenolic compound metabolism might increase (Michalak, 2006). This might lead to an increase in phenolic compounds and polyphenol derivatives, which could bind heavy metals, enhance the activity of antioxidant enzymes, and reduce the harmful effects of heavy metals on plants (Güez et al., 2017; Manquián-Cerdaa et al., 2018) (Figure 6). Therefore, in response to low-level (T1) and high-level (T3) Cd treatment, *S. miltiorrhiza* roots upregulate their secondary metabolism, mainly by upregulating eudesmic acid and rosmarinic acid to resist Cd stress (Figure 6) (Zoufan et al., 2020).

### Fatty Acids Play Vital Roles in Resisting Cd Stress

Our study proved that fatty acids played vital roles in resisting Cd stress. Compared to CK, most discriminating fatty acids (e.g., oleic acid and linoleic acid) decreased significantly both in T1 and T2, and unsaturated fatty acids (e.g., oleic acid and linoleic acid) decreased significantly, with all of the discriminating saturated fatty acids (e.g., stearic acid) being upregulated (Figure 5; Supplementary Table S2) in T3. As discussed above, in plants, fatty acids and their derivatives play vital roles in improving stress tolerance by participating in various defense pathways, including basal, systemic, and effector-triggered immunity (Chaffai et al., 2009; Liu et al., 2014; Li-Beisson et al., 2016). Unsaturated fatty acids (e.g., oleic acid and linoleic acid) are produced by the catalysis of fatty acid desaturases (FADs), which can catalyze the formation of double bonds at specific positions in the chain of saturated fatty acids (e.g., stearic acid) to regulate the response to various stresses (Liu et al., 2011b; Park et al., 2015). It is important to regulate the lipid composition and adjust the unsaturation level of membrane fatty acids to cope with metal stress (Thompson, 1992) (Figures 4, 6; Supplementary Table S2).

Therefore, oleic acid and linoleic acid, which could induce the production of plant reactive oxygen species, showed a decreased accumulation in T1, T2 and T3 to maintain the normal growth of *S. miltiorrhiza* under Cd stress (Cury-Boaventura and Curi, 2005) (Figure 5), and high-level Cd stress might inhibit the expression of FADs, leading to the enrichment of saturated fatty acids (especially stearic acid) (Figure 5) (Liu et al., 2013). In contrast to our study, other researchers found that Cd stress increased the contents of oleic acid, linoleic acid, and stearic acid in *Asterioneilu glacialis* (Jones et al., 1987) and decreased the linoleic acid content in *Sedum plumbizincicola* (Sun et al., 2020) and the oleic acid content in *Sedum alfredii* (Luo et al., 2014). As mentioned above, these differences might be caused by the differences in the Cd sensitivities of different plants (Zhang and Chen, 2021).

## Conclusion

Metabolic regulation is one of the vital mechanisms by which plants respond to various stresses. In this study, we characterized the roles of metabolic regulation in *S. miltiorrhiza* roots under different levels of Cd stress in a pot experiment. First, amino acids, organic acids, and fatty acids played essential roles in resisting Cd stress in *S. miltiorrhiza* roots due to their larger proportions than the other metabolites and their major contributions in distinguishing roots between the CK and each Cd treatment group (T1, T2, and T3). Moreover, biosynthesis of unsaturated fatty acids, isoquinoline alkaloid biosynthesis, betalain biosynthesis, aminoacyl-tRNA biosynthesis, and tyrosine metabolism played vital roles in Cd resistance due to their involvement in the synthesis of these metabolites. Second, amino acids, organic acids, and fatty acids, which play vital roles in resisting Cd stress, were different in *S. miltiorrhiza* roots under different levels of Cd stress. Because of the important roles of these metabolites in stress resistance, *S. miltiorrhiza* roots might mainly upregulate o-tyrosine, chorismite and eudesmic acid under low Cd (25 mg kg^−1^) stress; DL-tryptophan, L-aspartic acid, L-proline and chorismite under moderate Cd (50 mg kg^−1^) stress; and L-proline, L-serine, L-histidine, eudesmic acid, and rosmarinic acid under high Cd (100 mg kg^−1^) stress. *S. miltiorrhiza* mainly downregulated unsaturated fatty acids (e.g., oleic acid and linoleic acid) under all levels of Cd stress and upregulated saturated fatty acids (especially stearic acid) under high Cd stress.

## Data Availability Statement

The original contributions presented in the study are included in the article/Supplementary Material, further inquiries can be directed to the corresponding author/s.

## Author Contributions

JY, HF, and XW: conceptualization, writing-original draft preparation, writing-review and editing, and supervision. JY and HF: validation. RL and SS: investigation and project administration. JY, RL, and SS: resources. JY and XW: funding acquisition. All authors contributed to the article and approved the submitted version.

## Funding

This work was supported by the Scientific Research Foundation for Doctor of the Jiangxi University of Chinese Medicine (2020BSZR011); and the National Key R&D Program of China (2019YFC1712302).

## Conflict of Interest

The authors declare that the research was conducted in the absence of any commercial or financial relationships that could be construed as a potential conflict of interest.

## Publisher's Note

All claims expressed in this article are solely those of the authors and do not necessarily represent those of their affiliated organizations, or those of the publisher, the editors and the reviewers. Any product that may be evaluated in this article, or claim that may be made by its manufacturer, is not guaranteed or endorsed by the publisher.

## Supplementary Material

Supplementary Figure S1 Total ion chromatograms (TICs) with positive **(A)** and negative **(B)** ion modes of CK and (quality control) QC samples.

Supplementary Figure S2 Total ion chromatograms (TICs) with positive **(A)** and negative **(B)** ion modes of (quality control) QC samples.

Supplementary Figure S3 Permutation test results of OPLS-DA models for metabolomic data from *S. miltiorrhiza* roots with different levels of Cd stress. **(A–C)** represent the permutation test results of OPLS-DA models for *S. miltiorrhiza* roots of the control and 25 mg kg^−1^ Cd stress groups (R2Y [1] = 0.74, Q2 [1] = 0.99, CV-ANOVA *p* = 0.02), the control and 50 mg kg^−1^ Cd stress groups (R2Y [1] = 0.74, Q2 [1] = 0.99, CV-ANOVA *p* = 0.03) and the control and 100 mg kg^−1^ Cd stress groups (R2Y [1] = 0.81, Q2 [1] = 0.99, CV-ANOVA *p* = 0.01), respectively.

Supplementary Table S1 Primary and secondary mass spectrometry analysis conditions.

Supplementary Table S2 All metabolites identified by LC–MS/MS in *S. miltiorrhiza* roots with different levels of Cd stress. CK, T1, T2 and T3 represent roots in the control, 25 mg kg^−1^ Cd, 50 mg kg^−1^ Cd, and 100 mg kg^−1^ Cd treated groups (*n* = 3), respectively (the same below). All data are presented as the mean ± SE.

Supplementary Table S3 Relative contents of metabolites of different types in *S. miltiorrhiza* roots with different levels of Cd stress. CK, T1, T2 and T3 represent roots in the control, 25 mg kg^−1^ Cd, 50 mg kg^−1^ Cd, and 100 mg kg^−1^ Cd treated groups, respectively (the same below). Different lowercase letters indicate significant differences within each treatment in the roots (*p* < 0.05). All data are presented as the mean ± SE (*n* = 3).

Supplementary Table S4 Results of pathway analysis involving all of the discriminating metabolites in *S. miltiorrhiza* roots with different levels of Cd stress. All pathways shown in the table are potential target metabolic pathways with pathway impacts above 0.1; Total Cmpd, total number of compounds in the pathway; Hits, the number of actually matched compounds in the pathway; Holm adjust, *p* value adjusted by the Holm–Bonferroni method; FDR, *p* value adjusted using the False Discovery Rate; Impact, pathway impact value.
