# Supplementary figures and images for: Untargeted LC–MS/MS-Based Metabolomic Profiling for the Edible and Medicinal Plant Salvia miltiorrhiza Under Different Levels of Cadmium Stress
Source: Front Plant Sci. 2022 Jul 28;13:889370. doi: 10.3389/fpls.2022.889370 (PMC9366474; doi:10.3389/fpls.2022.889370)

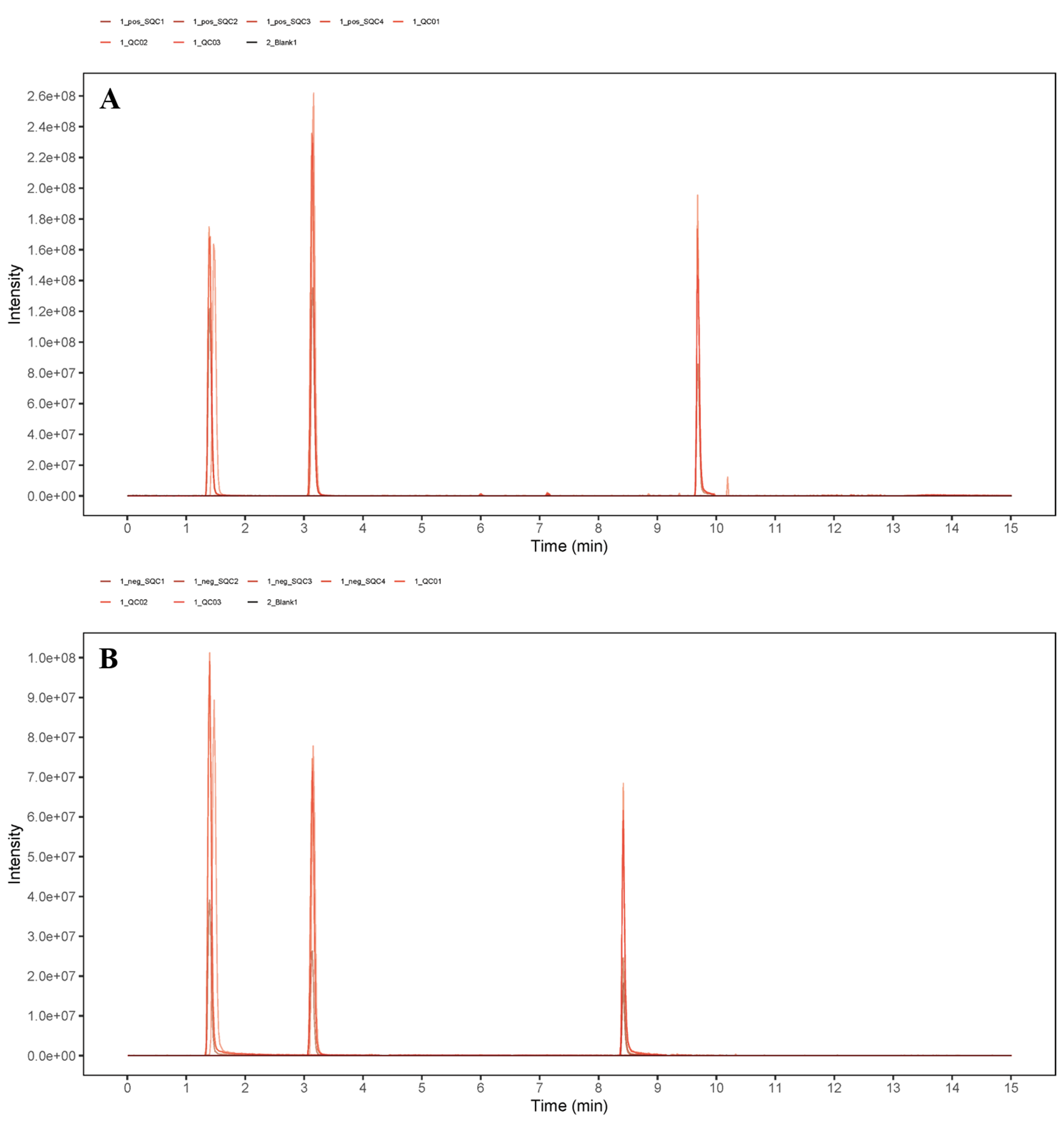

Supplement: Supplementary Figure S1 — Total ion chromatograms (TICs) with positive (A) and negative (B) ion modes of CK and (quality control) QC samples. [file Image_1.TIF]

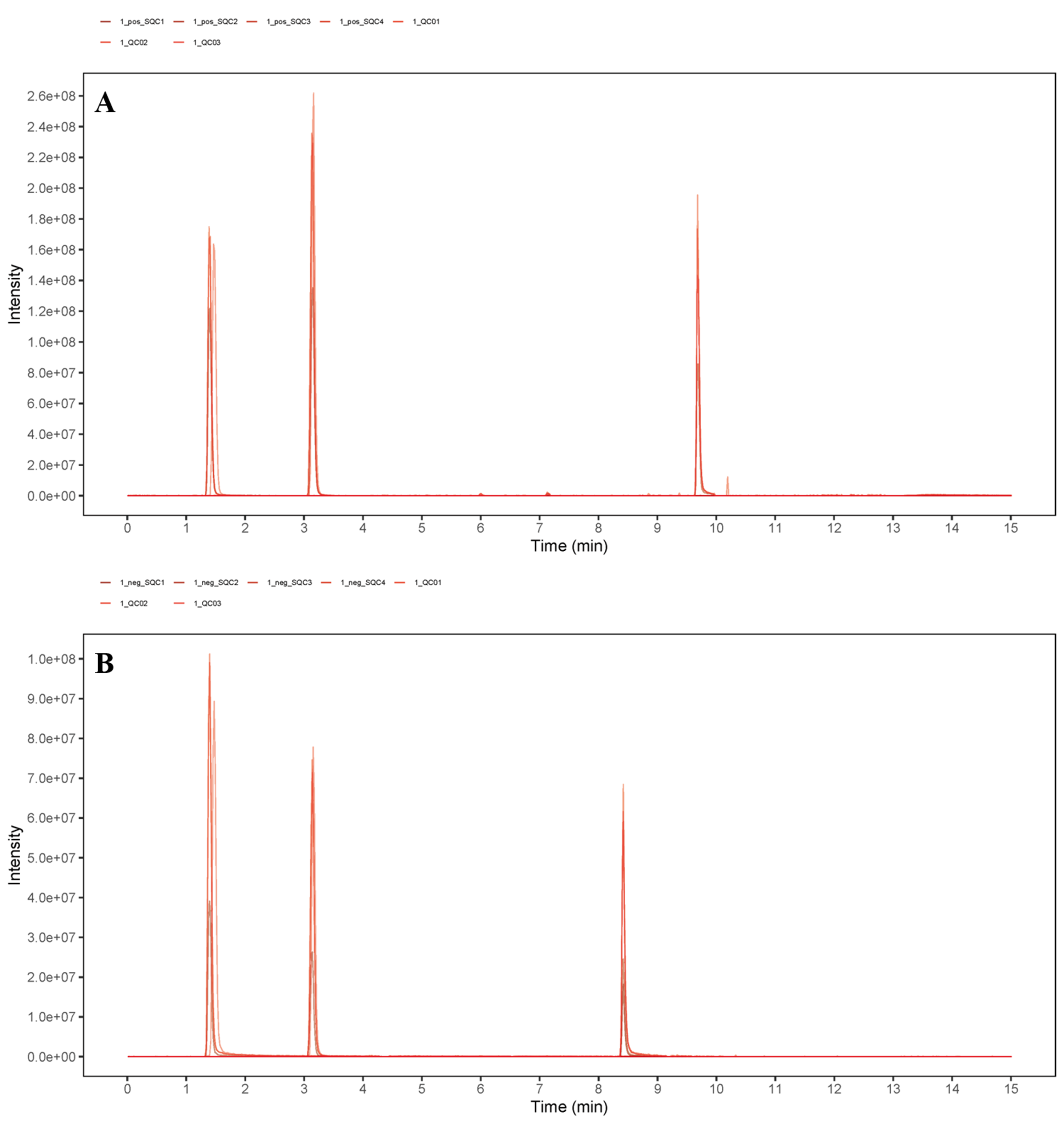

Supplement: Supplementary Figure S2 — Total ion chromatograms (TICs) with positive (A) and negative (B) ion modes of (quality control) QC samples. [file Image_2.TIF]

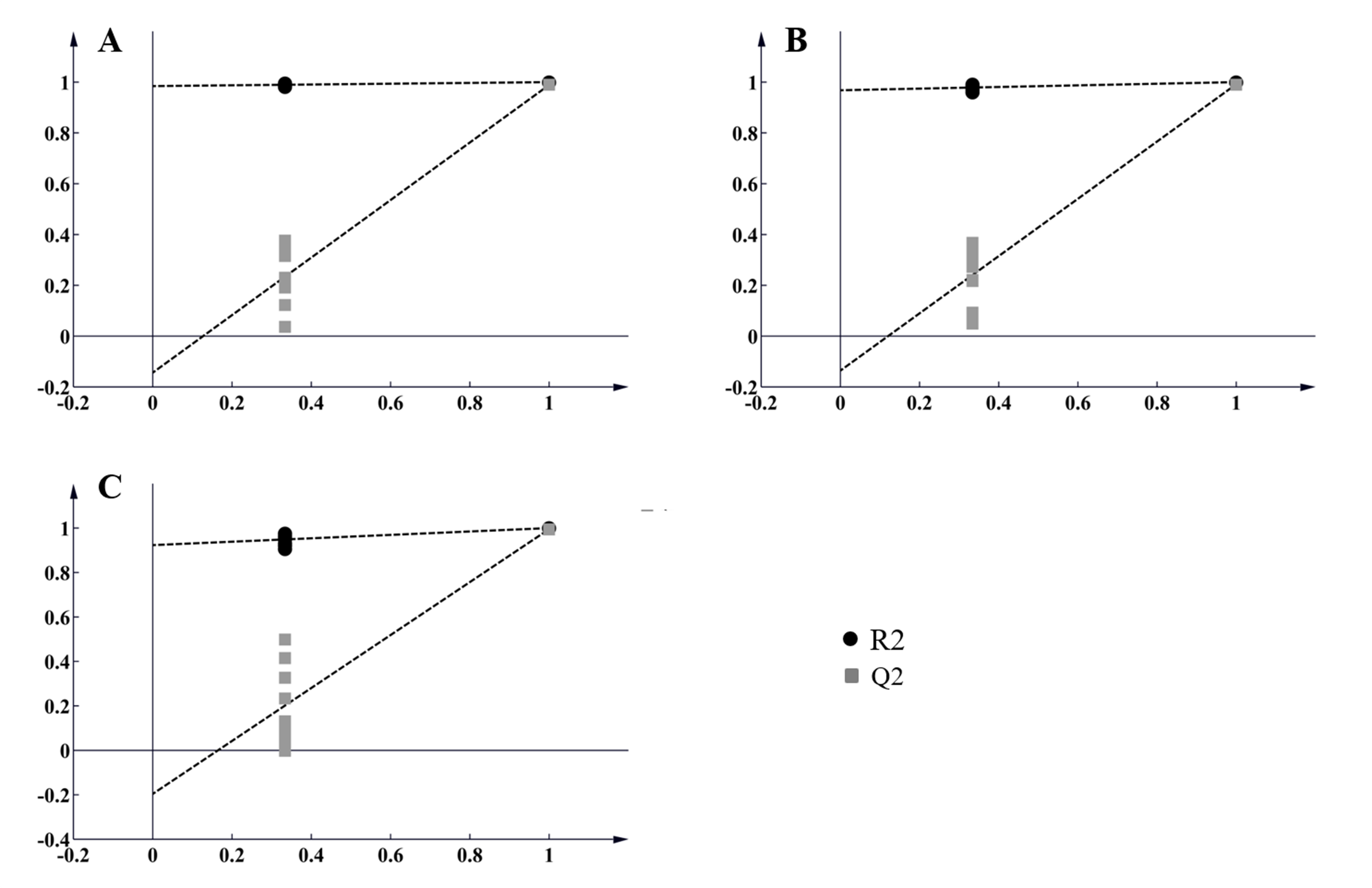

Supplement: Supplementary Figure S3 — Permutation test results of OPLS-DA models for metabolomic data from S. miltiorrhiza roots with different levels of Cd stress. (A–C) represent the permutation test results of OPLS-DA models for S. miltiorrhiza roots of the control and 25 mg kg−1 Cd stress groups (R2Y [1] = 0.74, Q2 [1] = 0.99, CV-ANOVA p = 0.02), the control and 50 mg kg−1 Cd stress groups (R2Y [1] = 0.74, Q2 [1] = 0.99, CV-ANOVA p = 0.03) and the control and 100 mg kg−1 Cd stress groups (R2Y [1] = 0.81, Q2 [1] = 0.99, CV-ANOVA p = 0.01), respectively. [file Image_3.TIF]
