# Supplementary material for: Untargeted LC–MS/MS-Based Metabolomic Profiling for the Edible and Medicinal Plant Salvia miltiorrhiza Under Different Levels of Cadmium Stress
Source: Front Plant Sci. 2022 Jul 28;13:889370. doi: 10.3389/fpls.2022.889370 (PMC9366474; doi:10.3389/fpls.2022.889370)
Supplement: Supplementary Table S1 — Primary and secondary mass spectrometry analysis conditions. [file Table_1.DOCX]

Supply table 1 Primary and secondary mass spectrometry analysis conditions.

| Types | Index | Conditions |
| --- | --- | --- |
| Primary mass spectrometry | AGC | 3e+6 |
|  | Scanning range | 70-1050 m/z |
|  | full MS resolution | [60000@m/z 200](mailto:60000@m/z200) |
|  | Maximum IT | 100 ms |
|  | capillary temperature | 350℃ |
|  | Runtime | 15 min |
| Secondary mass spectrometry | MS2 Activation Type | HCD |
|  | AGC target | 2e+5 |
|  | Isolation window | 1.5m/z |
|  | MS/MS resolution | 7500@m/z 200 |
|  | Maximum IT | 50 ms |
|  | collision energy | 10/30/60 |
