# Supplementary material for: Untargeted LC–MS/MS-Based Metabolomic Profiling for the Edible and Medicinal Plant Salvia miltiorrhiza Under Different Levels of Cadmium Stress
Source: Front Plant Sci. 2022 Jul 28;13:889370. doi: 10.3389/fpls.2022.889370 (PMC9366474; doi:10.3389/fpls.2022.889370)
Supplement: Supplementary Table S2 — All metabolites identified by LC–MS/MS in S. miltiorrhiza roots with different levels of Cd stress. CK, T1, T2 and T3 represent roots in the control, 25 mg kg−1 Cd, 50 mg kg−1 Cd, and 100 mg kg−1 Cd treated groups (n = 3), respectively (the same below). All data are presented as the mean ± SE. [file Table_2.DOCX]

Supply table 2 All metabolites identified by LC–MS/MS in *S. miltiorrhiza* roots with different levels of Cd stress.

| Metabolites | RT (s) | Mass | CK |  | T1 | | |  | T2 | | |  | T3 | | |
| --- | --- | --- | --- | --- | --- | --- | --- | --- | --- | --- | --- | --- | --- | --- | --- |
|  |  |  | Mean ± SE |  | Mean ± SE | *p* | VIP |  | Mean ± SE | *p* | VIP |  | Mean ± SE | *p* | VIP |
| Gibberellin A3 | 331.20 | 345.14 | 104.88 ± 1.78 |  | 4.97 ± 0.10 | 0.00 | 1.24 |  | 35.72 ± 0.91 | > 0.05 | <1 |  | 7.89 ± 0.48 | 0.00 | <1 |
| Cohumulone | 421.61 | 347.19 | 99.76 ± 38.39 |  | 105.63 ± 30.30 | 0.00 | <1 |  | 71.49 ± 31.66 | > 0.05 | <1 |  | 222.10 ± 92.49 | 0.00 | 1.16 |
| Isokobusone | 441.09 | 221.15 | 73.25 ± 0.17 |  | 27.71 ± 0.42 | 0.00 | 1.24 |  | 66.46 ± 1.43 | > 0.05 | <1 |  | 49.43 ± 0.39 | 0.00 | <1 |
| Inosine | 40.55 | 267.07 | 56.31 ± 0.69 |  | 29.64 ± 0.28 | > 0.05 | 1.24 |  | 49.66 ± 0.84 | 0.02 | 1.11 |  | 71.44 ± 1.28 | 0.00 | 1.16 |
| 2',4',6'-Trihydroxyacetophenone | 218.06 | 167.03 | 49.67 ± 0.24 |  | 27.46 ± 0.38 | 0.00 | 1.24 |  | 27.33 ± 0.37 | 0.02 | 1.12 |  | 49.00 ± 1.96 | 0.00 | 1.16 |
| Mycophenolic acid | 419.34 | 319.12 | 17.72 ± 0.10 |  | 2.15 ± 0.09 | 0.00 | 1.24 |  | 11.35 ± 0.59 | 0.01 | 1.17 |  | 1.87 ± 0.03 | 0.00 | 1.15 |
| 13-OxoODE | 552.03 | 293.21 | 15.40 ± 1.68 |  | 28.39 ± 0.32 | 0.00 | 1.20 |  | 9.33 ± 0.76 | 0.00 | 1.25 |  | 0.51 ± 0.18 | 0.00 | 1.10 |
| L-Erythrulose | 45.05 | 119.03 | 15.26 ± 0.24 |  | 11.66 ± 0.22 | 0.06 | 1.22 |  | 10.96 ± 0.15 | > 0.05 | <1 |  | 17.20 ± 0.34 | 0.00 | 1.04 |
| Glucobrassicin | 240.42 | 447.06 | 13.62 ± 0.07 |  | 1.95 ± 0.04 | 0.00 | 1.24 |  | 8.32 ± 0.06 | > 0.05 | <1 |  | 14.98 ± 0.27 | > 0.05 | 1.16 |
| Methylgingerol | 466.25 | 307.19 | 12.73 ± 0.28 |  | 6.28 ± 0.03 | 0.00 | 1.23 |  | 8.40 ± 0.28 | > 0.05 | <1 |  | 12.55 ± 0.75 | 0.00 | 1.04 |
| D-Glutamine | 43.92 | 145.06 | 10.38 ± 0.39 |  | 12.47 ± 0.08 | 0.00 | 1.16 |  | 35.93 ± 1.26 | 0.00 | 1.20 |  | 62.23 ± 2.28 | 0.00 | 1.09 |
| Undecanoic acid | 540.53 | 185.15 | 10.23 ± 0.21 |  | 1.50 ± 0.04 | > 0.05 | 1.24 |  | 2.79 ± 0.06 | 0.02 | 1.12 |  | 4.71 ± 0.09 | 0.00 | 1.14 |
| Methylsuccinic acid | 72.42 | 131.03 | 9.12 ± 0.90 |  | 13.70 ± 1.93 | > 0.05 | < 1 |  | 15.37 ± 1.79 | > 0.05 | < 1 |  | 29.41 ± 6.22 | > 0.05 | <1 |
| 15-Deoxy-d-12,14-PGJ2 | 475.01 | 315.20 | 8.84 ± 0.09 |  | 9.76 ± 0.06 | 0.00 | 1.21 |  | 14.08 ± 0.18 | 0.00 | 1.25 |  | 18.59 ± 0.19 | 0.00 | 1.09 |
| 3-Hydroxypicolinic acid | 793.11 | 138.02 | 8.69 ± 0.36 |  | 2.42 ± 0.08 | > 0.05 | 1.23 |  | 4.37 ± 0.12 | > 0.05 | <1 |  | 9.80 ± 0.25 | > 0.05 | 1.08 |
| α-Linolenic acid | 480.07 | 277.22 | 8.66 ± 1.53 |  | 1.90 ± 0.03 | 0.00 | 1.13 |  | 5.47 ± 0.12 | 0.00 | 1.25 |  | 3.19 ± 0.18 | > 0.05 | <1 |
| Resolvin D2 | 473.92 | 375.22 | 7.45 ± 2.75 |  | 5.39 ± 0.12 | > 0.05 | <1 |  | 6.54 ± 0.39 | 0.00 | 1.23 |  | 18.99 ± 2.71 | > 0.05 | 1.16 |
| Isopalmitic acid | 579.89 | 255.23 | 7.05 ± 0.08 |  | 1.26 ± 0.02 | > 0.05 | 1.24 |  | 3.55 ± 0.06 | 0.00 | 1.21 |  | 5.12 ± 0.12 | 0.00 | <1 |
| 3,4-Dihydroxyhydrocinnamic acid | 231.58 | 181.05 | 6.88 ± 2.40 |  | 6.88 ± 0.06 | 0.00 | <1 |  | 10.55 ± 0.41 | 0.00 | 1.25 |  | 12.64 ± 0.22 | 0.00 | 1.14 |
| Hypoxanthine | 742.04 | 135.03 | 6.41 ± 0.16 |  | 3.95 ± 0.41 | > 0.05 | 1.17 |  | 6.27 ± 0.23 | > 0.05 | <1 |  | 6.17 ± 0.27 | 0.04 | 1.16 |
| (S,E)-Zearalenone | 361.18 | 317.14 | 6.15 ± 0.08 |  | 3.46 ± 0.04 | 0.00 | 1.24 |  | 8.03 ± 0.05 | > 0.05 | <1 |  | 6.16 ± 0.07 | 0.04 | 1.06 |
| D-2,3-Dihydroxypropanoic acid | 41.69 | 105.02 | 5.96 ± 0.77 |  | 7.02 ± 0.55 | > 0.05 | <1 |  | 7.60 ± 0.68 | > 0.05 | <1 |  | 4.24 ± 0.02 | 0.02 | <1 |
| Nicotinic acid | 842.09 | 122.02 | 5.79 ± 0.06 |  | 17.21 ± 0.43 | > 0.05 | 1.24 |  | 18.57 ± 0.22 | > 0.05 | <1 |  | 13.37 ± 0.12 | > 0.05 | <1 |
| Estriol | 506.47 | 287.17 | 5.46 ± 0.26 |  | 2.57 ± 0.06 | 0.00 | 1.22 |  | 5.11 ± 0.02 | 0.00 | 1.25 |  | 5.67 ± 0.08 | 0.02 | <1 |
| Ethyl glucuronide | 18.37 | 221.07 | 5.28 ± 2.16 |  | 5.82 ± 1.92 | > 0.05 | <1 |  | 5.43 ± 1.57 | 0.00 | 1.23 |  | 11.67 ± 3.35 | 0.02 | 1.14 |
| Corticosterone | 330.03 | 381.18 | 4.78 ± 0.14 |  | 11.40 ± 0.16 | 0.00 | 1.24 |  | 8.97 ± 0.23 | 0.00 | 1.23 |  | 12.47 ± 0.12 | 0.00 | <1 |
| [10]-Dehydrogingerdione | 529.10 | 345.21 | 4.69 ± 0.06 |  | 4.16 ± 0.09 | 0.00 | 1.15 |  | 6.33 ± 0.15 | 0.00 | 1.22 |  | 7.07 ± 0.21 | 0.00 | 1.16 |
| 1,3,7-Trimethyluric acid | 43.92 | 209.07 | 4.58 ± 0.63 |  | 7.85 ± 1.39 | 0.00 | <1 |  | 7.83 ± 0.64 | 0.00 | 1.23 |  | 15.63 ± 2.01 | 0.00 | 1.15 |
| Maslinic acid | 595.16 | 471.35 | 4.39 ± 0.88 |  | 3.30 ± 0.84 | 0.00 | <1 |  | 2.25 ± 0.28 | 0.00 | 1.26 |  | 8.86 ± 3.55 | > 0.05 | 1.13 |
| Levoglucosan | 874.26 | 161.05 | 4.11 ± 0.02 |  | 5.03 ± 0.09 | > 0.05 | 1.22 |  | 6.11 ± 0.07 | > 0.05 | <1 |  | 5.94 ± 0.14 | > 0.05 | <1 |
| M-toluic Acid | 798.98 | 135.04 | 4.07 ± 0.14 |  | 0.78 ± 0.02 | > 0.05 | 1.23 |  | 1.57 ± 0.06 | > 0.05 | <1 |  | 1.97 ± 0.07 | > 0.05 | 1.16 |
| Palmitic acid | 562.25 | 255.23 | 3.81 ± 0.04 |  | 3.16 ± 0.04 | 0.01 | 1.22 |  | 17.47 ± 1.84 | 0.00 | 1.20 |  | 16.88 ± 2.18 | 0.00 | 1.14 |
| Estrone | 559.95 | 269.15 | 3.79 ± 0.08 |  | 0.87 ± 0.01 | 0.00 | 1.24 |  | 2.51 ± 0.12 | 0.00 | 1.24 |  | 0.80 ± 0.06 | 0.01 | <1 |
| 8,15-DiHETE | 490.54 | 335.22 | 3.76 ± 0.11 |  | 0.52 ± 0.00 | > 0.05 | 1.24 |  | 1.97 ± 0.19 | 0.02 | 1.12 |  | 1.22 ± 0.03 | > 0.05 | <1 |
| Steviobioside | 575.33 | 641.31 | 3.68 ± 0.05 |  | 0.89 ± 0.01 | 0.00 | 1.24 |  | 2.08 ± 0.10 | > 0.05 | <1 |  | 1.01 ± 0.08 | 0.00 | 1.14 |
| Ethyl stearate | 610.43 | 311.30 | 3.62 ± 0.13 |  | 5.23 ± 0.13 | > 0.05 | 1.21 |  | 6.74 ± 0.24 | > 0.05 | <1 |  | 4.64 ± 0.12 | > 0.05 | 1.16 |
| L-Glutamic acid | 41.67 | 146.05 | 3.46 ± 0.41 |  | 8.20 ± 2.97 | > 0.05 | <1 |  | 2.95 ± 0.10 | > 0.05 | <1 |  | 6.57 ± 0.63 | > 0.05 | 1.15 |
| Cytosine | 651.65 | 110.04 | 3.40 ± 0.06 |  | 0.75 ± 0.02 | > 0.05 | 1.24 |  | 2.22 ± 0.06 | > 0.05 | <1 |  | 0.72 ± 0.05 | > 0.05 | 1.16 |
| L-Aspartic acid | 40.55 | 132.03 | 3.25 ± 0.06 |  | 0.70 ± 0.01 | 0.01 | 1.24 |  | 1.49 ± 0.02 | 0.00 | 1.24 |  | 1.84 ± 0.01 | 0.00 | 1.15 |
| 3-Methoxyanthranilate | 249.94 | 166.05 | 3.19 ± 0.49 |  | 5.24 ± 1.07 | 0.01 | <1 |  | 6.37 ± 3.19 | 0.00 | <1 |  | 6.97 ± 0.68 | 0.00 | <1 |
| Adenine | 160.26 | 134.05 | 3.11 ± 0.14 |  | 1.39 ± 0.01 | > 0.05 | 1.22 |  | 3.07 ± 0.26 | > 0.05 | <1 |  | 2.69 ± 0.17 | > 0.05 | <1 |
| Methyl vanillate | 312.27 | 181.05 | 2.98 ± 0.01 |  | 2.89 ± 0.04 | > 0.05 | <1 |  | 2.92 ± 0.05 | > 0.05 | <1 |  | 4.11 ± 0.04 | 0.01 | <1 |
| 5-HEPE | 523.37 | 317.21 | 2.88 ± 0.04 |  | 0.63 ± 0.17 | 0.00 | 1.23 |  | 2.27 ± 0.05 | 0.00 | 1.25 |  | 1.06 ± 0.32 | 0.00 | 1.15 |
| 3-Methyl-2-oxovaleric acid | 840.64 | 129.06 | 2.43 ± 0.03 |  | 4.55 ± 0.12 | 0.02 | 1.23 |  | 7.32 ± 0.18 | 0.02 | 1.10 |  | 4.66 ± 0.14 | 0.00 | 1.13 |
| Monoethyl phthalate | 347.26 | 193.05 | 2.39 ± 0.07 |  | 3.60 ± 0.11 | 0.00 | 1.21 |  | 4.44 ± 0.48 | 0.00 | 1.20 |  | 7.38 ± 0.07 | 0.00 | <1 |
| Umbelliferone | 264.98 | 161.02 | 2.38 ± 0.99 |  | 2.00 ± 0.11 | 0.02 |  |  | 1.86 ± 0.07 | 0.00 | 1.26 |  | 6.09 ± 0.74 | 0.00 | 1.02 |
| Gluconic acid | 41.69 | 195.05 | 2.32 ± 0.04 |  | 2.58 ± 0.05 | 0.00 | 1.10 |  | 7.38 ± 0.14 | > 0.05 | <1 |  | 13.56 ± 0.09 | > 0.05 | 1.15 |
| Caprylic acid | 430.68 | 143.11 | 2.32 ± 0.04 |  | 3.12 ± 0.09 | > 0.05 | 1.20 |  | 4.56 ± 0.08 | > 0.05 | <1 |  | 7.09 ± 0.18 | 0.00 | 1.12 |
| DL-Dopa | 85.53 | 196.06 | 2.31 ± 0.03 |  | 3.24 ± 0.65 | 0.00 | <1 |  | 2.95 ± 0.23 | 0.00 | 1.26 |  | 6.56 ± 0.52 | > 0.05 | <1 |
| [6]-Dehydrogingerdione | 428.88 | 289.15 | 2.30 ± 0.20 |  | 3.58 ± 0.11 | 0.01 | 1.17 |  | 1.98 ± 0.17 | 0.00 | 1.20 |  | 1.05 ± 0.91 | > 0.05 | 1.16 |
| LysoPA(16:0/0:0) | 512.17 | 409.24 | 2.30 ± 0.02 |  | 1.69 ± 0.04 | 0.01 | 1.23 |  | 1.18 ± 0.11 | > 0.05 | <1 |  | 3.05 ± 0.22 | 0.00 | <1 |
| Dodecanoic acid | 570.17 | 199.17 | 2.11 ± 0.41 |  | 1.86 ± 0.36 | 0.01 | <1 |  | 2.00 ± 0.31 | 0.00 | 1.19 |  | 3.47 ± 0.19 | 0.00 | 1.03 |
| 2,3-Dihydroxybutanedioic acid | 581.51 | 149.01 | 2.04 ± 0.06 |  | 3.15 ± 0.02 | > 0.05 | 1.23 |  | 7.01 ± 0.04 | > 0.05 | <1 |  | 3.27 ± 0.04 | 0.00 | 1.16 |
| Isoferulic acid | 289.41 | 193.05 | 2.03 ± 0.06 |  | 0.40 ± 0.01 | 0.00 | 1.24 |  | 2.32 ± 0.05 | 0.03 | 1.08 |  | 0.73 ± 0.01 | 0.01 | 1.15 |
| 2',5,6-Trimethoxyflavone | 524.41 | 311.09 | 2.00 ± 0.87 |  | 0.91 ± 0.06 | 0.00 | <1 |  | 1.21 ± 0.02 | > 0.05 | <1 |  | 3.54 ± 1.26 | > 0.05 | 1.14 |
| 3α-Acetoxy-11-keto-β-boswellic acid | 611.61 | 511.34 | 1.92 ± 0.06 |  | 0.80 ± 0.01 | > 0.05 | 1.23 |  | 1.55 ± 0.19 | > 0.05 | <1 |  | 1.82 ± 0.20 | > 0.05 | 1.14 |
| Thyrotropin releasing hormone | 510.70 | 361.17 | 1.83 ± 0.34 |  | 2.75 ± 0.74 | 0.03 | <1 |  | 2.31 ± 0.25 | > 0.05 | <1 |  | 4.61 ± 0.70 | > 0.05 | 1.03 |
| Vanillin | 99.43 | 151.04 | 1.82 ± 0.51 |  | 1.12 ± 0.18 | 0.00 | <1 |  | 0.88 ± 0.05 | 0.01 | 1.14 |  | 2.08 ± 0.08 | 0.02 | 1.14 |
| 5-Methylcytosine | 779.30 | 124.05 | 1.67 ± 0.28 |  | 2.08 ± 0.31 | > 0.05 | <1 |  | 1.57 ± 0.13 | > 0.05 | <1 |  | 3.59 ± 0.65 | 0.00 | 1.14 |
| Myristic acid | 624.58 | 227.20 | 1.63 ± 0.07 |  | 0.94 ± 0.07 | 0.04 | 1.19 |  | 1.85 ± 0.10 | > 0.05 | <1 |  | 0.78 ± 0.01 | 0.00 | <1 |
| Dimethylmalonic acid | 870.47 | 131.03 | 1.55 ± 0.03 |  | 1.63 ± 0.01 | 0.72 | <1 |  | 1.52 ± 0.05 | > 0.05 | <1 |  | 3.11 ± 0.05 | 0.03 | 1.16 |
| 5,7-dihydroxy-2-(4-methoxyphenyl)-3,4-dihydro-2H-1-benzopyran-4-one | 242.75 | 285.08 | 1.51 ± 0.02 |  | 1.63 ± 0.10 | 0.00 | <1 |  | 2.43 ± 0.61 | 0.00 | 1.26 |  | 3.90 ± 0.18 | 0.00 | <1 |
| Citraconic acid | 49.54 | 129.02 | 1.47 ± 0.21 |  | 1.73 ± 0.51 | 0.29 | <1 |  | 3.52 ± 1.03 | > 0.05 | <1 |  | 2.70 ± 0.54 | 0.00 | 1.15 |
| Quercetin 3-O-(6''-acetyl-glucoside) | 253.54 | 505.10 | 1.44 ± 0.01 |  | 0.79 ± 0.01 | 0.00 | 1.24 |  | 1.11 ± 0.04 | > 0.05 | <1 |  | 1.37 ± 0.01 | 0.00 | <1 |
| Ethyl hexadecanoate | 495.89 | 283.26 | 1.38 ± 0.03 |  | 1.16 ± 0.02 | 0.59 | 1.18 |  | 1.78 ± 0.06 | 0.01 | 1.15 |  | 2.06 ± 0.01 | 0.01 | <1 |
| Enterodiol | 296.64 | 301.14 | 1.30 ± 0.38 |  | 0.63 ± 0.20 | 0.07 | <1 |  | 0.37 ± 0.16 | > 0.05 | <1 |  | 0.27 ± 0.05 | > 0.05 | 1.15 |
| 20-Hydroxyeicosatetraenoic acid | 496.13 | 319.23 | 1.26 ± 0.24 |  | 1.75 ± 0.48 | 0.00 | <1 |  | 1.15 ± 0.19 | 0.00 | 1.26 |  | 3.12 ± 0.92 | 0.00 | 1.15 |
| 3-Hydroxybenzoic acid | 833.28 | 137.02 | 1.24 ± 0.03 |  | 1.10 ± 0.04 | 0.88 | 1.05 |  | 1.31 ± 0.04 | > 0.05 | <1 |  | 2.14 ± 0.09 | > 0.05 | 1.07 |
| 3-Hydroxyphenylacetic acid | 864.30 | 151.04 | 1.16 ± 0.01 |  | 0.48 ± 0.00 | 0.81 | 1.24 |  | 1.17 ± 0.04 | > 0.05 | <1 |  | 1.02 ± 0.04 | > 0.05 | <1 |
| (R)-lipoic acid | 786.74 | 223.02 | 1.13 ± 0.00 |  | 0.63 ± 0.01 | 0.84 | 1.24 |  | 1.20 ± 0.04 | > 0.05 | <1 |  | 1.15 ± 0.05 | 0.00 | 1.13 |
| Suberic acid | 623.12 | 173.08 | 1.02 ± 0.03 |  | 0.64 ± 0.01 | 0.01 | 1.23 |  | 1.07 ± 0.04 | > 0.05 | <1 |  | 1.33 ± 0.02 | 0.00 | 1.14 |
| 3-Furoic acid | 784.86 | 111.01 | 1.01 ± 0.04 |  | 0.77 ± 0.02 | 0.14 | 1.17 |  | 0.51 ± 0.00 | 0.00 | 1.19 |  | 1.15 ± 0.01 | 0.00 | 1.06 |
| Eicosapentaenoic acid | 579.33 | 301.22 | 1.00 ± 0.15 |  | 0.98 ± 0.36 | 0.00 | <1 |  | 0.95 ± 0.09 | > 0.05 | <1 |  | 2.64 ± 1.02 | > 0.05 | 1.15 |
| (2E)-3-(2-hydroxyphenyl)-1-(4-methoxyphenyl)prop-2-en-1-one | 547.45 | 253.09 | 0.98 ± 0.17 |  | 1.37 ± 0.20 | 0.00 | <1 |  | 1.40 ± 0.07 | 0.01 | 1.17 |  | 2.96 ± 1.11 | > 0.05 | 1.03 |
| Pelargonic acid | 475.99 | 157.12 | 0.96 ± 0.28 |  | 0.62 ± 0.02 | 0.08 | <1 |  | 0.41 ± 0.04 | > 0.05 | <1 |  | 1.45 ± 0.13 | 0.00 | 1.05 |
| Hydroxyphenyllactic acid | 267.32 | 181.05 | 0.94 ± 0.04 |  | 1.09 ± 0.24 | 0.00 | <1 |  | 1.59 ± 0.26 | 0.00 | 1.26 |  | 3.84 ± 0.72 | 0.00 | 1.13 |
| (-)-Matairesinol | 445.96 | 357.13 | 0.94 ± 0.13 |  | 1.06 ± 0.16 | 0.00 | <1 |  | 0.74 ± 0.09 | 0.01 | 1.18 |  | 3.08 ± 0.88 | 0.02 | 1.15 |
| γ-Aminobutyric acid | 41.67 | 102.06 | 0.92 ± 0.03 |  | 1.09 ± 0.01 | 0.00 | 1.15 |  | 1.13 ± 0.01 | > 0.05 | <1 |  | 2.04 ± 0.05 | 0.00 | 1.01 |
| 1,7-bis(4-hydroxyphenyl)-5-methoxyheptan-3-one | 546.33 | 327.16 | 0.88 ± 0.19 |  | 2.23 ± 0.56 | 0.00 | <1 |  | 3.14 ± 0.73 | 0.00 | 1.23 |  | 1.91 ± 0.16 | 0.00 | <1 |
| Fertaric acid | 247.56 | 325.06 | 0.86 ± 0.01 |  | 0.88 ± 0.03 | 0.36 | <1 |  | 0.68 ± 0.04 | > 0.05 | <1 |  | 1.89 ± 0.11 | > 0.05 | <1 |
| 3-Carboxy-4-methyl-5-propyl-2-furanpropionic acid | 246.75 | 239.09 | 0.85 ± 0.01 |  | 0.43 ± 0.01 | 0.00 | 1.24 |  | 1.29 ± 0.15 | 0.01 | 1.16 |  | 0.73 ± 0.04 | > 0.05 | <1 |
| L-Serine | 59.80 | 104.03 | 0.84 ± 0.21 |  | 0.43 ± 0.05 | 0.01 | <1 |  | 0.49 ± 0.04 | 0.00 | 1.24 |  | 1.12 ± 0.17 | 0.02 | 1.14 |
| Genipin | 298.21 | 225.08 | 0.80 ± 0.01 |  | 1.04 ± 0.03 | 0.00 | 1.20 |  | 1.33 ± 0.03 | 0.02 | 1.12 |  | 0.97 ± 0.03 | 0.00 | 1.15 |
| Maltopentaose | 71.35 | 827.27 | 0.76 ± 0.02 |  | 0.15 ± 0.00 | 0.00 | <1 |  | 0.45 ± 0.01 | > 0.05 | <1 |  | 0.66 ± 0.02 | 0.04 | 1.16 |
| Phenobarbital | 223.47 | 231.08 | 0.75 ± 0.08 |  | 0.92 ± 0.07 | 0.06 | <1 |  | 0.72 ± 0.05 | 0.00 | 1.25 |  | 1.57 ± 0.21 | 0.01 | 1.16 |
| 1,11-Undecanedicarboxylic acid | 265.87 | 243.16 | 0.72 ± 0.02 |  | 0.67 ± 0.04 | 0.00 | <1 |  | 0.52 ± 0.01 | 0.01 | 1.14 |  | 1.18 ± 0.08 | 0.00 | 1.08 |
| Sucrose | 60.84 | 341.11 | 0.71 ± 0.00 |  | 0.26 ± 0.00 | 0.31 | 1.24 |  | 0.54 ± 0.08 | > 0.05 | <1 |  | 0.38 ± 0.01 | > 0.05 | 1.16 |
| 5-Aminopentanoic acid | 50.71 | 116.07 | 0.71 ± 0.06 |  | 0.62 ± 0.06 | 0.00 | <1 |  | 0.41 ± 0.13 | 0.00 | 1.26 |  | 1.53 ± 0.29 | 0.00 | 1.14 |
| Bovinic acid | 644.94 | 279.23 | 0.70 ± 0.03 |  | 0.10 ± 0.00 | 0.00 | 1.23 |  | 0.44 ± 0.09 | 0.00 | 1.26 |  | 0.31 ± 0.01 | 0.00 | 1.15 |
| Arachidonic acid | 597.43 | 303.23 | 0.69 ± 0.01 |  | 0.72 ± 0.02 | 0.00 | <1 |  | 0.76 ± 0.01 | > 0.05 | <1 |  | 1.47 ± 0.04 | 0.01 | 1.02 |
| o-Tyrosine | 190.68 | 180.07 | 0.69 ± 0.04 |  | 1.23 ± 0.05 | 0.00 | 1.20 |  | 2.19 ± 0.16 | 0.00 | 1.25 |  | 1.40 ± 0.68 | 0.00 | <1 |
| Tetrahydrocurcumin | 476.12 | 371.15 | 0.68 ± 0.03 |  | 0.21 ± 0.01 | 0.05 | 1.23 |  | 0.23 ± 0.01 | 0.00 | 1.23 |  | 0.37 ± 0.03 | > 0.05 | 1.04 |
| 2,6-Pyridinedicarboxylic acid | 743.15 | 166.02 | 0.67 ± 0.01 |  | 0.17 ± 0.00 | 0.56 | 1.24 |  | 0.23 ± 0.01 | > 0.05 | <1 |  | 0.71 ± 0.02 | 0.01 | <1 |
| βmipron | 190.68 | 192.07 | 0.66 ± 0.06 |  | 0.85 ± 0.04 | 0.00 | <1 |  | 0.68 ± 0.02 | 0.00 | 1.25 |  | 1.61 ± 0.07 | 0.00 | 1.13 |
| 3-(4-hydroxy-3-methoxyphenyl)prop-2-enoic acid | 247.71 | 193.05 | 0.66 ± 0.12 |  | 0.50 ± 0.07 | 0.03 | <1 |  | 0.46 ± 0.01 | 0.00 | 1.20 |  | 0.81 ± 0.03 | > 0.05 | <1 |
| Esculentic acid (Diplazium) | 514.09 | 487.34 | 0.64 ± 0.02 |  | 0.46 ± 0.02 | 0.00 | 1.19 |  | 0.48 ± 0.05 | 0.00 | 1.26 |  | 0.66 ± 0.01 | 0.00 | 1.14 |
| Acetylglycine |  |  | 0.60 ± 0.03 |  | 0.74 ± 0.11 | #DIV/0! | <1 |  | 0.55 ± 0.16 | > 0.05 | <1 |  | 1.11 ± 0.03 | #DIV/0! | 1.14 |
| N,N'-Diacetylhydrazine | 10.72 | 115.04 | 0.59 ± 0.00 |  | 0.65 ± 0.03 | 0.11 | <1 |  | 0.73 ± 0.04 | > 0.05 | <1 |  | 0.95 ± 0.03 | > 0.05 | <1 |
| 11-Dehydrocorticosterone | 518.99 | 343.19 | 0.57 ± 0.02 |  | 1.57 ± 0.05 | 0.00 | 1.23 |  | 2.68 ± 0.10 | 0.04 | 1.04 |  | 0.73 ± 0.20 | 0.00 | 1.16 |
| Benazeprilat | 325.34 | 395.16 | 0.56 ± 0.08 |  | 0.66 ± 0.04 | 0.00 | <1 |  | 0.31 ± 0.15 | 0.00 | 1.25 |  | 1.51 ± 0.55 | 0.00 | 1.06 |
| Aesculetin | 265.02 | 177.02 | 0.55 ± 0.03 |  | 0.40 ± 0.01 | 0.01 | 1.16 |  | 0.41 ± 0.02 | 0.00 | 1.23 |  | 0.68 ± 0.03 | > 0.05 | 1.14 |
| 4-[(E)-2-(3,5-dimethoxyphenyl)ethenyl]phenol | 449.98 | 255.10 | 0.54 ± 0.02 |  | 0.70 ± 0.06 | 0.00 | <1 |  | 1.33 ± 0.06 | > 0.05 | <1 |  | 3.27 ± 2.30 | 0.00 | <1 |
| Dihydrouracil | 891.28 | 113.02 | 0.54 ± 0.26 |  | 0.28 ± 0.04 | 0.36 | <1 |  | 0.16 ± 0.01 | > 0.05 | <1 |  | 0.49 ± 0.08 | > 0.05 | 1.15 |
| D-Malic acid | 223.37 | 133.01 | 0.53 ± 0.01 |  | 0.77 ± 0.01 | 0.11 | 1.23 |  | 0.76 ± 0.02 | 0.03 | 1.18 |  | 0.94 ± 0.01 | 0.00 | 1.12 |
| Glucose 6-phosphate | 39.43 | 259.02 | 0.53 ± 0.01 |  | 0.58 ± 0.01 | 0.01 | 1.09 |  | 0.57 ± 0.01 | 0.01 | 1.13 |  | 1.37 ± 0.02 | 0.03 | 1.15 |
| 11(R)-HETE | 524.65 | 319.23 | 0.52 ± 0.04 |  | 0.52 ± 0.00 | 0.00 | <1 |  | 0.52 ± 0.01 | 0.00 | 1.25 |  | 1.03 ± 0.03 | 0.00 | 1.08 |
| Fumaric acid | 72.20 | 115.00 | 0.52 ± 0.02 |  | 0.85 ± 0.13 | 0.06 | <1 |  | 0.49 ± 0.02 | > 0.05 | <1 |  | 0.90 ± 0.03 | 0.00 | 1.05 |
| Dihydrojasmonic acid | 485.81 | 211.13 | 0.52 ± 0.01 |  | 0.31 ± 0.01 | 0.02 | 1.22 |  | 0.23 ± 0.03 | > 0.05 | <1 |  | 0.26 ± 0.00 | 0.00 | 1.12 |
| Carnosol | 545.75 | 329.18 | 0.51 ± 0.01 |  | 0.23 ± 0.00 | 0.00 | 1.24 |  | 0.94 ± 0.01 | 0.04 | 1.04 |  | 0.32 ± 0.00 | 0.04 | 1.11 |
| Secoisolariciresinol | 443.09 | 361.17 | 0.49 ± 0.06 |  | 0.41 ± 0.01 | 0.00 | <1 |  | 0.37 ± 0.02 | > 0.05 | <1 |  | 0.41 ± 0.05 | > 0.05 | 1.13 |
| Gibberellin A53 | 489.23 | 347.19 | 0.48 ± 0.16 |  | 0.43 ± 0.01 | 0.02 | <1 |  | 0.15 ± 0.03 | > 0.05 | <1 |  | 0.55 ± 0.22 | 0.03 | <1 |
| Estrone sulfate | 229.47 | 349.11 | 0.48 ± 0.03 |  | 0.42 ± 0.01 | 0.00 | <1 |  | 0.65 ± 0.07 | 0.00 | 1.25 |  | 1.14 ± 0.06 | 0.00 | <1 |
| γ-CEHC | 314.97 | 263.13 | 0.47 ± 0.11 |  | 0.24 ± 0.02 | 0.00 | <1 |  | 0.31 ± 0.05 | > 0.05 | <1 |  | 0.81 ± 0.10 | 0.00 | <1 |
| (ÃÂ±)-Tryptophan | 240.37 | 203.08 | 0.45 ± 0.01 |  | 0.43 ± 0.01 | 0.46 | <1 |  | 0.52 ± 0.02 | 0.01 | 1.15 |  | 0.88 ± 0.03 | > 0.05 | 1.15 |
| Azelaic acid | 304.31 | 187.10 | 0.45 ± 0.03 |  | 0.47 ± 0.02 | 0.37 | <1 |  | 0.57 ± 0.07 | 0.00 | 1.24 |  | 1.13 ± 0.09 | 0.00 | 1.14 |
| 3-Hydroxyflavone | 383.36 | 237.06 | 0.44 ± 0.02 |  | 0.53 ± 0.02 | 0.00 | 1.05 |  | 0.57 ± 0.02 | 0.01 | 1.14 |  | 0.79 ± 0.07 | 0.00 | 1.09 |
| L-Asparagine | 42.81 | 131.05 | 0.44 ± 0.00 |  | 0.57 ± 0.00 | 0.00 | 1.24 |  | 0.40 ± 0.01 | 0.00 | 1.25 |  | 0.65 ± 0.01 | > 0.05 | 1.03 |
| Butylparaben | 439.93 | 193.09 | 0.43 ± 0.04 |  | A ± 0.00 | 0.02 | <1 |  | 0.38 ± 0.01 | > 0.05 | <1 |  | A ± 0.00 | 0.00 | <1 |
| Leukotriene B5 | 472.61 | 333.21 | 0.42 ± 0.01 |  | 0.45 ± 0.01 | 0.00 | <1 |  | 0.39 ± 0.01 | 0.00 | 1.23 |  | 0.64 ± 0.06 | 0.00 | 1.14 |
| D-Xylulose | 776.89 | 149.05 | 0.41 ± 0.08 |  | 0.33 ± 0.01 | 0.08 | <1 |  | 0.17 ± 0.02 | 0.04 | 1.05 |  | 0.65 ± 0.07 | 0.01 | 1.07 |
| 4-Dodecylbenzenesulfonic Acid | 504.93 | 325.18 | 0.41 ± 0.03 |  | 0.56 ± 0.02 | 0.10 | 1.10 |  | 0.58 ± 0.01 | > 0.05 | <1 |  | 0.73 ± 0.01 | 0.02 | <1 |
| L-Tyrosine | 51.83 | 180.07 | 0.40 ± 0.13 |  | 0.42 ± 0.15 | 0.00 | <1 |  | 0.37 ± 0.04 | 0.00 | 1.25 |  | 0.53 ± 0.09 | > 0.05 | 1.16 |
| Pyruvic acid | 780.42 | 87.01 | 0.40 ± 0.01 |  | 0.49 ± 0.01 | 0.56 | 1.19 |  | 0.39 ± 0.01 | 0.01 | 1.15 |  | 0.99 ± 0.01 | 0.00 | 1.04 |
| 12-HEPE | 581.45 | 317.21 | 0.40 ± 0.04 |  | 0.39 ± 0.09 | 0.00 | <1 |  | 0.44 ± 0.13 | > 0.05 | <1 |  | 1.23 ± 0.36 | 0.02 | <1 |
| L-Lactic acid | 45.04 | 89.02 | 0.40 ± 0.03 |  | 0.40 ± 0.01 | 0.66 | <1 |  | 0.25 ± 0.00 | > 0.05 | <1 |  | 0.85 ± 0.22 | 0.04 | 1.15 |
| Adipic acid | 625.77 | 145.05 | 0.40 ± 0.02 |  | 0.11 ± 0.00 | 0.41 | 1.22 |  | 0.30 ± 0.02 | > 0.05 | <1 |  | 0.20 ± 0.00 | > 0.05 | 1.09 |
| Xanthoxylin | 287.25 | 195.07 | 0.40 ± 0.01 |  | 0.05 ± 0.00 | 0.89 | 1.24 |  | 0.09 ± 0.00 | 0.00 | <1 |  | 0.10 ± 0.00 | 0.00 | 1.13 |
| L-Arginine | 205.98 | 173.10 | 0.39 ± 0.01 |  | 0.46 ± 0.01 | 0.00 | 1.18 |  | 0.55 ± 0.01 | 0.00 | 1.26 |  | 0.11 ± 0.03 | 0.00 | <1 |
| Mono-benzyl phthalate | 398.08 | 255.07 | 0.39 ± 0.05 |  | 0.73 ± 0.02 | 0.00 | 1.19 |  | 0.33 ± 0.02 | 0.00 | 1.19 |  | 0.14 ± 0.07 | 0.00 | 1.09 |
| Imidazoleacetic acid | 194.17 | 125.04 | 0.38 ± 0.08 |  | 0.07 ± 0.00 | 0.00 | 1.11 |  | 0.29 ± 0.01 | 0.00 | 1.25 |  | 0.13 ± 0.00 | 0.00 | 1.11 |
| Myricetin 3-neohesperidoside | 353.05 | 625.14 | 0.37 ± 0.08 |  | 0.39 ± 0.12 | 0.29 | <1 |  | 0.19 ± 0.01 | > 0.05 | <1 |  | 0.46 ± 0.04 | 0.00 | 1.15 |
| Caffeic acid | 260.46 | 179.03 | 0.37 ± 0.09 |  | 0.23 ± 0.03 | 0.00 | <1 |  | 0.22 ± 0.04 | 0.00 | 1.25 |  | 0.46 ± 0.11 | 0.00 | <1 |
| Demethoxycurcumin | 435.20 | 337.11 | 0.35 ± 0.00 |  | 0.41 ± 0.01 | 0.51 | 1.17 |  | 0.32 ± 0.00 | > 0.05 | <1 |  | 0.29 ± 0.01 | > 0.05 | 1.09 |
| L-Norleucine | 83.58 | 130.09 | 0.35 ± 0.01 |  | 0.47 ± 0.03 | 0.00 | 1.11 |  | 0.44 ± 0.01 | 0.00 | 1.24 |  | 0.83 ± 0.05 | 0.00 | 1.05 |
| Glaucarubin | 317.26 | 495.22 | 0.34 ± 0.01 |  | 0.09 ± 0.01 | 0.29 | 1.23 |  | 0.15 ± 0.01 | 0.00 | 1.25 |  | 0.23 ± 0.02 | 0.01 | 1.15 |
| Ethyl dodecanoate | 532.61 | 227.20 | 0.32 ± 0.02 |  | 0.31 ± 0.02 | 0.13 | <1 |  | 0.19 ± 0.01 | 0.04 | <1 |  | 0.74 ± 0.17 | 0.00 | 1.15 |
| 2-Methylbenzoic acid | 260.46 | 135.04 | 0.31 ± 0.02 |  | 0.29 ± 0.04 | 0.00 | <1 |  | 0.36 ± 0.01 | 0.01 | 1.14 |  | 0.68 ± 0.03 | 0.00 | 1.13 |
| Citric acid | 387.85 | 191.02 | 0.31 ± 0.01 |  | 0.36 ± 0.03 | 0.00 | <1 |  | 0.23 ± 0.01 | 0.03 | <1 |  | 0.60 ± 0.04 | > 0.05 | <1 |
| Terephthalic acid | 222.22 | 165.02 | 0.30 ± 0.01 |  | 0.26 ± 0.00 | 0.36 | 1.04 |  | 0.62 ± 0.03 | 0.00 | 1.24 |  | 0.86 ± 0.04 | 0.10 | <1 |
| L-Proline | 47.33 | 114.06 | 0.30 ± 0.00 |  | 0.35 ± 0.00 | 0.00 | 1.22 |  | 0.11 ± 0.03 | 0.00 | 1.22 |  | 0.55 ± 0.16 | 0.00 | 1.06 |
| Stachyose | 72.40 | 665.22 | 0.29 ± 0.01 |  | 0.31 ± 0.07 | 0.00 | <1 |  | 0.31 ± 0.04 | 0.03 | 1.08 |  | 0.41 ± 0.03 | 0.00 | 1.12 |
| Tridecanoic acid | 552.59 | 213.19 | 0.29 ± 0.01 |  | 0.27 ± 0.00 | 0.42 | <1 |  | 0.27 ± 0.00 | > 0.05 | <1 |  | 0.52 ± 0.02 | 0.02 | <1 |
| Kojic acid | 730.74 | 141.02 | 0.28 ± 0.00 |  | 0.06 ± 0.00 | 0.49 | 1.24 |  | 0.42 ± 0.01 | > 0.05 | <1 |  | 0.37 ± 0.01 | 0.04 | 1.03 |
| β-Alanine | 42.79 | 88.04 | 0.27 ± 0.01 |  | 0.86 ± 0.22 | 0.00 | 1.00 |  | 0.52 ± 0.06 | 0.00 | 1.25 |  | 0.83 ± 0.12 | 0.00 | <1 |
| Phenylethyl primeveroside | 229.96 | 461.17 | 0.27 ± 0.01 |  | 0.31 ± 0.04 | 0.49 | <1 |  | 0.37 ± 0.01 | > 0.05 | <1 |  | 0.68 ± 0.20 | 0.02 | <1 |
| Oleic acid | 676.56 | 281.25 | 0.27 ± 0.02 |  | 0.14 ± 0.00 | 0.00 | 1.17 |  | 0.14 ± 0.01 | 0.00 | 1.25 |  | 0.18 ± 0.01 | 0.00 | 1.14 |
| Aflatoxin B1 | 332.37 | 311.06 | 0.27 ± 0.06 |  | 0.25 ± 0.03 | 0.00 | <1 |  | 0.13 ± 0.00 | 0.03 | <1 |  | 0.41 ± 0.03 | 0.00 | 1.16 |
| 2-Furoylglycine | 746.50 | 168.03 | 0.26 ± 0.11 |  | 0.32 ± 0.02 | 0.31 | <1 |  | 0.29 ± 0.02 | > 0.05 | <1 |  | 0.37 ± 0.03 | 0.02 | 1.16 |
| L-Phenylalanine | 190.68 | 164.07 | 0.26 ± 0.03 |  | 0.31 ± 0.02 | 0.00 | <1 |  | 0.21 ± 0.04 | 0.00 | 1.26 |  | 0.39 ± 0.06 | 0.00 | <1 |
| p-Anisic acid | 245.57 | 151.04 | 0.26 ± 0.01 |  | 0.10 ± 0.00 | 0.02 | 1.23 |  | 0.20 ± 0.01 | 0.01 | 1.18 |  | 0.07 ± 0.01 | 0.00 | 1.15 |
| Glycocholic acid | 360.93 | 464.30 | 0.26 ± 0.02 |  | 0.24 ± 0.03 | 0.55 | <1 |  | 0.21 ± 0.02 | > 0.05 | <1 |  | 0.44 ± 0.03 | > 0.05 | 1.04 |
| Vanillic acid | 410.03 | 167.03 | 0.25 ± 0.01 |  | 0.36 ± 0.02 | 0.21 | 1.15 |  | 0.28 ± 0.02 | > 0.05 | <1 |  | 0.73 ± 0.02 | 0.04 | 1.13 |
| bicyclo-PGE2 | 397.14 | 333.21 | 0.25 ± 0.00 |  | 0.38 ± 0.00 | 0.00 | 1.23 |  | 0.49 ± 0.00 | 0.01 | 1.17 |  | 0.63 ± 0.01 | 0.00 | 1.15 |
| Eupatilin | 278.89 | 343.08 | 0.24 ± 0.04 |  | 0.36 ± 0.01 | 0.00 | 1.05 |  | 0.48 ± 0.01 | 0.00 | 1.26 |  | 0.33 ± 0.01 | 0.00 | <1 |
| Prostaglandin D3 | 463.29 | 349.20 | 0.24 ± 0.00 |  | 0.04 ± 0.00 | 0.00 | 1.24 |  | 0.15 ± 0.00 | > 0.05 | <1 |  | 0.09 ± 0.00 | 0.00 | <1 |
| 16-Hydroxy hexadecanoic acid | 558.86 | 271.23 | 0.24 ± 0.02 |  | 0.34 ± 0.10 | 0.46 |  |  | 0.24 ± 0.01 | > 0.05 | <1 |  | 0.53 ± 0.01 | 0.01 | 1.14 |
| Trans-3-coumarate | 241.20 | 163.04 | 0.24 ± 0.01 |  | 0.26 ± 0.01 | 0.02 |  |  | 0.15 ± 0.01 | > 0.05 | <1 |  | 0.37 ± 0.03 | > 0.05 | <1 |
| γ-Glutamylmethionine | 575.97 | 277.09 | 0.23 ± 0.01 |  | 0.25 ± 0.00 | 0.00 |  |  | 0.26 ± 0.01 | > 0.05 | <1 |  | 0.50 ± 0.02 | 0.00 | 1.16 |
| Oxytetracycline | 52.96 | 459.14 | 0.23 ± 0.00 |  | 0.03 ± 0.01 | 0.20 | 1.23 |  | 0.15 ± 0.00 | > 0.05 | <1 |  | 0.07 ± 0.00 | > 0.05 | <1 |
| 4',5,7-Trihydroxy-6-prenylflavanone | 482.01 | 339.12 | 0.22 ± 0.00 |  | 0.29 ± 0.07 | 0.00 | <1 |  | 0.20 ± 0.00 | 0.00 | 1.25 |  | 0.38 ± 0.02 | 0.00 | 1.12 |
| Meconine | 321.96 | 193.05 | 0.22 ± 0.00 |  | 0.29 ± 0.03 | 0.00 | <1 |  | 0.12 ± 0.01 | 0.04 | 1.03 |  | 0.59 ± 0.03 | 0.00 | <1 |
| Succinic anhydride | 54.09 | 99.01 | 0.22 ± 0.04 |  | 0.35 ± 0.13 | 0.00 | <1 |  | 0.14 ± 0.02 | 0.00 | 1.24 |  | 0.56 ± 0.20 | 0.03 | <1 |
| 2-Methylglutaric acid | 769.13 | 145.05 | 0.22 ± 0.01 |  | 0.09 ± 0.00 | 0.91 | 1.23 |  | 0.24 ± 0.01 | > 0.05 | <1 |  | 0.23 ± 0.03 | > 0.05 | <1 |
| Propionic acid | 353.85 | 73.03 | 0.21 ± 0.01 |  | 0.05 ± 0.00 | 0.37 | 1.23 |  | 0.17 ± 0.00 | > 0.05 | <1 |  | 0.07 ± 0.00 | 0.00 | 1.13 |
| Ursolic acid | 644.94 | 455.35 | 0.20 ± 0.03 |  | 0.23 ± 0.05 | 0.00 | <1 |  | 0.14 ± 0.01 | 0.00 | 1.24 |  | 0.50 ± 0.07 | > 0.05 | 1.14 |
| Pentadecanoic acid | 641.51 | 241.22 | 0.20 ± 0.00 |  | 0.26 ± 0.00 | 0.17 | 1.24 |  | 0.28 ± 0.02 | 0.05 | 1.02 |  | 0.42 ± 0.06 | 0.00 | <1 |
| 2-Oxovaleric acid | 796.59 | 115.04 | 0.19 ± 0.07 |  | 0.17 ± 0.05 | 0.94 | <1 |  | 0.09 ± 0.00 | > 0.05 | <1 |  | 0.27 ± 0.01 | > 0.05 | <1 |
| Heptanoic acid | 402.86 | 129.09 | 0.19 ± 0.01 |  | 0.55 ± 0.01 | 0.26 | 1.24 |  | 0.54 ± 0.03 | > 0.05 | <1 |  | 1.56 ± 0.00 | 0.00 | <1 |
| Methyl 2-hydroxybenzoate | 392.50 | 151.04 | 0.19 ± 0.00 |  | 0.01 ± 0.00 | 0.22 | 1.24 |  | 0.07 ± 0.00 | 0.03 | <1 |  | 0.18 ± 0.00 | 0.00 | 1.10 |
| 4-Pyridoxic acid | 761.17 | 182.05 | 0.19 ± 0.00 |  | 0.25 ± 0.01 | 0.09 | 1.19 |  | 0.29 ± 0.01 | 0.02 | 1.11 |  | 0.47 ± 0.01 | 0.01 | 1.14 |
| Pyridoxal | 79.05 | 166.05 | 0.18 ± 0.02 |  | 0.24 ± 0.04 | 0.04 |  |  | 0.14 ± 0.06 | > 0.05 | <1 |  | 0.31 ± 0.06 | > 0.05 | 1.07 |
| 3-Methyladipic acid | 659.05 | 159.07 | 0.17 ± 0.00 |  | 0.01 ± 0.00 | 0.18 | 1.24 |  | 0.06 ± 0.00 | > 0.05 | <1 |  | 0.06 ± 0.00 | 0.01 | <1 |
| Daidzin | 415.80 | 461.11 | 0.17 ± 0.01 |  | 0.22 ± 0.01 | 0.96 | 1.10 |  | 0.14 ± 0.00 | > 0.05 | <1 |  | 0.43 ± 0.03 | 0.00 | 1.00 |
| 2-Ketobutyric acid | 599.70 | 101.02 | 0.17 ± 0.03 |  | 0.05 ± 0.00 | 0.34 | 1.12 |  | 0.08 ± 0.01 | 0.04 | 1.04 |  | 0.11 ± 0.00 | > 0.05 | <1 |
| Levocetirizine | 401.71 | 387.15 | 0.17 ± 0.03 |  | 0.21 ± 0.04 | 0.00 | <1 |  | 0.13 ± 0.00 | 0.00 | 1.24 |  | 0.37 ± 0.03 | 0.00 | 1.04 |
| Hypogeic acid | 638.03 | 253.22 | 0.17 ± 0.05 |  | 0.19 ± 0.04 | 0.13 | <1 |  | 0.14 ± 0.01 | > 0.05 | <1 |  | 0.32 ± 0.01 | > 0.05 | <1 |
| 1-Kestose | 49.52 | 503.16 | 0.16 ± 0.01 |  | 0.14 ± 0.01 | 0.00 | <1 |  | 0.19 ± 0.00 | 0.00 | 1.25 |  | 0.25 ± 0.01 | 0.01 | 1.10 |
| Apiin | 237.07 | 563.14 | 0.15 ± 0.03 |  | 0.04 ± 0.00 | 0.00 | 1.08 |  | 0.13 ± 0.00 | 0.00 | 1.21 |  | 0.05 ± 0.00 | 0.00 | <1 |
| Norbuprenorphine | 455.34 | 412.25 | 0.15 ± 0.00 |  | 0.13 ± 0.00 | 0.14 | 1.18 |  | 0.23 ± 0.00 | > 0.05 | <1 |  | 0.11 ± 0.01 | > 0.05 | <1 |
| 15-Methylpalmitate | 693.49 | 269.25 | 0.15 ± 0.00 |  | 0.19 ± 0.00 | 0.00 | 1.20 |  | 0.15 ± 0.00 | > 0.05 | <1 |  | 0.21 ± 0.00 | > 0.05 | <1 |
| 2-Hydroxyestradiol | 454.05 | 287.17 | 0.15 ± 0.00 |  | 0.18 ± 0.01 | 0.00 | 1.05 |  | 0.13 ± 0.01 | 0.00 | 1.24 |  | 0.37 ± 0.08 | 0.00 | <1 |
| Pyroglutamic acid | 795.44 | 128.03 | 0.15 ± 0.00 |  | 0.21 ± 0.01 | 0.36 | 1.18 |  | 0.18 ± 0.01 | > 0.05 | <1 |  | 0.20 ± 0.01 | > 0.05 | <1 |
| Porphobilinogen | 681.68 | 225.09 | 0.15 ± 0.00 |  | 0.47 ± 0.00 | 0.15 | 1.24 |  | 0.57 ± 0.02 | > 0.05 | <1 |  | 0.31 ± 0.01 | 0.01 | <1 |
| Medicagenic acid | 458.68 | 501.32 | 0.15 ± 0.00 |  | 0.03 ± 0.00 | 0.01 | 1.23 |  | 0.15 ± 0.01 | 0.01 | 1.16 |  | 0.03 ± 0.00 | > 0.05 | 1.07 |
| Cortisone | 483.62 | 359.19 | 0.15 ± 0.03 |  | 0.10 ± 0.02 | 0.01 | <1 |  | 0.13 ± 0.02 | 0.05 | 1.03 |  | 0.21 ± 0.02 | > 0.05 | 1.08 |
| 2,2-Dimethylsuccinic acid | 14.82 | 145.05 | 0.15 ± 0.00 |  | 0.37 ± 0.01 | 0.18 | 1.23 |  | 0.76 ± 0.00 | > 0.05 | <1 |  | 0.29 ± 0.08 | 0.00 | <1 |
| 3-Hydroxymethylglutaric acid | 809.52 | 161.05 | 0.14 ± 0.01 |  | 0.17 ± 0.01 | 0.23 | <1 |  | 0.37 ± 0.02 | > 0.05 | <1 |  | 0.46 ± 0.06 | > 0.05 | <1 |
| Quinic acid | 809.19 | 191.06 | 0.14 ± 0.00 |  | 0.01 ± 0.00 | 0.14 | 1.24 |  | 0.07 ± 0.00 | > 0.05 | <1 |  | 0.03 ± 0.00 | 0.00 | <1 |
| Theophylline | 68.37 | 179.06 | 0.14 ± 0.01 |  | 0.14 ± 0.01 | 0.63 | <1 |  | 0.24 ± 0.00 | > 0.05 | <1 |  | 0.06 ± 0.01 | > 0.05 | <1 |
| Oxoadipic acid | 48.45 | 159.03 | 0.14 ± 0.00 |  | 0.11 ± 0.01 | 0.18 | 1.00 |  | 0.10 ± 0.00 | > 0.05 | <1 |  | 0.20 ± 0.00 | 0.02 | <1 |
| Estradiol | 520.12 | 271.17 | 0.14 ± 0.00 |  | 0.06 ± 0.00 | 0.00 | 1.24 |  | 0.16 ± 0.00 | 0.00 | 1.26 |  | 0.19 ± 0.01 | 0.01 | <1 |
| Indoleacetaldehyde | 392.51 | 158.06 | 0.13 ± 0.01 |  | 0.13 ± 0.01 | 0.00 | <1 |  | 0.16 ± 0.00 | 0.00 | 1.26 |  | 0.30 ± 0.02 | 0.00 | <1 |
| 5,7-dihydroxy-2-(4-hydroxyphenyl)-6,8-dimethyl-3,4-dihydro-2H-1-benzopyran-4-one | 306.63 | 299.09 | 0.13 ± 0.00 |  | 0.06 ± 0.00 | 0.00 | 1.23 |  | 0.16 ± 0.01 | 0.04 | <1 |  | 0.07 ± 0.00 | 0.00 | <1 |
| 2-Furoic acid | 50.63 | 111.01 | 0.13 ± 0.00 |  | 0.15 ± 0.00 | 0.34 | 1.15 |  | 0.09 ± 0.01 | 0.00 | 1.23 |  | 0.34 ± 0.01 | 0.00 | <1 |
| Geranylgeranyl-PP | 315.01 | 449.18 | 0.11 ± 0.00 |  | 0.13 ± 0.00 | 0.00 | <1 |  | 0.08 ± 0.00 | 0.00 | 1.23 |  | 0.29 ± 0.01 | 0.04 | <1 |
| Syringic acid | 78.19 | 197.05 | 0.11 ± 0.00 |  | 0.06 ± 0.00 | 0.00 | 1.22 |  | 0.08 ± 0.00 | 0.00 | 1.23 |  | 0.04 ± 0.00 | > 0.05 | <1 |
| γ-Glutamylcysteine | 231.36 | 249.05 | 0.11 ± 0.00 |  | 0.13 ± 0.02 | 0.00 | <1 |  | 0.12 ± 0.01 | 0.00 | 1.25 |  | 0.26 ± 0.04 | 0.00 | <1 |
| Glutaric acid | 843.09 | 131.03 | 0.11 ± 0.00 |  | 0.02 ± 0.00 | 0.98 | 1.24 |  | 0.10 ± 0.00 | > 0.05 | <1 |  | 0.03 ± 0.00 | 0.01 | <1 |
| 16(17)-EpDPE | 639.27 | 343.23 | 0.11 ± 0.01 |  | 0.02 ± 0.00 | 0.37 | 1.23 |  | 0.09 ± 0.00 | > 0.05 | <1 |  | 0.04 ± 0.00 | 0.00 | <1 |
| L-Malic acid | 558.81 | 133.01 | 0.11 ± 0.01 |  | 0.11 ± 0.01 | 0.00 | <1 |  | 0.09 ± 0.00 | 0.00 | 1.24 |  | 0.19 ± 0.00 | 0.03 | <1 |
| 2-Methoxyestradiol | 622.92 | 301.18 | 0.10 ± 0.00 |  | 0.07 ± 0.00 | 0.30 | 1.17 |  | 0.09 ± 0.00 | 0.10 | <1 |  | 0.08 ± 0.00 | 0.05 | <1 |
| Pyrrolidonecarboxylic acid | 46.20 | 128.03 | 0.10 ± 0.00 |  | 0.10 ± 0.01 | 0.00 | <1 |  | 0.08 ± 0.00 | > 0.05 | <1 |  | 0.19 ± 0.01 | > 0.05 | <1 |
| Aspalathin | 299.74 | 451.13 | 0.10 ± 0.01 |  | 0.04 ± 0.01 | 0.01 | 1.16 |  | 0.09 ± 0.00 | 0.00 | 1.23 |  | 0.07 ± 0.01 | 0.00 | 1.11 |
| Glyceraldehyde | 804.26 | 89.02 | 0.10 ± 0.00 |  | 0.05 ± 0.01 | 0.06 | 1.20 |  | 0.09 ± 0.01 | 0.00 | 1.24 |  | 0.08 ± 0.01 | > 0.05 | <1 |
| Methyl jasmonate | 450.71 | 223.13 | 0.09 ± 0.02 |  | 0.11 ± 0.00 | 0.51 | <1 |  | 0.01 ± 0.00 | 0.00 | 1.26 |  | 0.31 ± 0.03 | 0.01 | <1 |
| Capric acid | 496.11 | 171.14 | 0.09 ± 0.00 |  | 0.03 ± 0.00 | 0.29 | 1.23 |  | 0.09 ± 0.00 | > 0.05 | <1 |  | 0.05 ± 0.00 | > 0.05 | 1.02 |
| Dehydroabietic acid | 605.61 | 299.20 | 0.09 ± 0.02 |  | 0.11 ± 0.02 | 0.01 | <1 |  | 0.12 ± 0.01 | > 0.05 | <1 |  | 0.19 ± 0.01 | 0.02 | <1 |
| Threonic acid | 657.36 | 135.03 | 0.08 ± 0.00 |  | 0.07 ± 0.00 | 0.27 | 1.08 |  | 0.07 ± 0.00 | > 0.05 | <1 |  | 0.05 ± 0.00 | > 0.05 | <1 |
| 8-iso-PGA1 | 469.00 | 335.22 | 0.08 ± 0.00 |  | 0.07 ± 0.00 | 0.00 | <1 |  | 0.03 ± 0.01 | > 0.05 | <1 |  | 0.02 ± 0.01 | 0.03 | 1.04 |
| Itaconic acid | 84.04 | 129.02 | 0.08 ± 0.00 |  | 0.05 ± 0.00 | 0.17 | 1.23 |  | 0.05 ± 0.00 | 0.00 | 1.20 |  | 0.03 ± 0.00 | 0.01 | <1 |
| Succinic acid | 54.03 | 117.02 | 0.08 ± 0.01 |  | 0.12 ± 0.00 | 0.00 | 1.14 |  | 0.15 ± 0.00 | 0.00 | 1.26 |  | 0.23 ± 0.07 | > 0.05 | <1 |
| L-Histidine | 43.94 | 154.06 | 0.08 ± 0.01 |  | 0.04 ± 0.00 | 0.04 | 1.02 |  | 0.05 ± 0.01 | 0.00 | 1.24 |  | 0.04 ± 0.01 | 0.00 | 1.02 |
| Cholesterol sulfate | 609.47 | 465.31 | 0.07 ± 0.01 |  | 0.08 ± 0.01 | 0.01 | <1 |  | 0.10 ± 0.02 | 0.01 | 1.15 |  | 0.20 ± 0.01 | 0.03 | <1 |
| Resolvin D1 | 507.69 | 375.22 | 0.07 ± 0.01 |  | 0.04 ± 0.00 | 0.00 | 1.06 |  | 0.03 ± 0.01 | 0.00 | 1.26 |  | 0.05 ± 0.00 | 0.00 | 1.13 |
| 18R-HEPE | 550.89 | 317.20 | 0.07 ± 0.00 |  | 0.02 ± 0.00 | 0.00 | 1.23 |  | 0.09 ± 0.00 | 0.00 | 1.24 |  | 0.03 ± 0.00 | 0.00 | <1 |
| 1,2,3-Trihydroxybenzene | 275.42 | 125.02 | 0.07 ± 0.00 |  | 0.13 ± 0.01 | 0.05 | 1.11 |  | 0.13 ± 0.03 | 0.00 | 1.22 |  | 0.09 ± 0.01 | 0.00 | <1 |
| Succinylacetone | 713.80 | 157.05 | 0.06 ± 0.00 |  | 0.20 ± 0.14 | 0.09 | <1 |  | 0.72 ± 0.61 | 0.03 | <1 |  | 0.01 ± 0.00 | 0.03 | <1 |
| Gibberellin A44 | 443.75 | 345.17 | 0.06 ± 0.00 |  | 0.04 ± 0.00 | 0.00 | 1.22 |  | 0.09 ± 0.01 | 0.04 | 1.05 |  | 0.08 ± 0.00 | 0.00 | <1 |
| Glycine | 42.81 | 74.02 | 0.06 ± 0.00 |  | 0.01 ± 0.00 | 0.14 | 1.21 |  | 0.07 ± 0.00 | > 0.05 | <1 |  | 0.05 ± 0.00 | 0.00 | 1.08 |
| cis,cis-Muconic acid | 669.79 | 141.02 | 0.06 ± 0.01 |  | 0.08 ± 0.01 | 0.65 | <1 |  | 0.09 ± 0.01 | > 0.05 | <1 |  | 0.05 ± 0.00 | > 0.05 | <1 |
| Rosmarinic acid | 264.98 | 359.08 | 0.06 ± 0.00 |  | 0.05 ± 0.01 | 0.01 | <1 |  | 0.06 ± 0.00 | 0.00 | 1.25 |  | 0.08 ± 0.00 | 0.00 | 1.15 |
| 9-cis-Retinoic acid | 553.15 | 299.20 | 0.05 ± 0.00 |  | 0.02 ± 0.00 | 0.00 | 1.22 |  | 0.05 ± 0.00 | 0.01 | 1.16 |  | 0.01 ± 0.00 | 0.00 | <1 |
| (R)-2-Benzylsuccinate | 285.92 | 207.07 | 0.05 ± 0.00 |  | 0.00 ± 0.00 | 0.03 | 1.23 |  | 0.02 ± 0.00 | 0.01 | 1.15 |  | 0.01 ± 0.00 | 0.00 | 1.12 |
| Citramalic acid | 49.60 | 147.03 | 0.05 ± 0.00 |  | 0.07 ± 0.01 | 0.00 |  |  | 0.06 ± 0.00 | 0.00 | 1.24 |  | 0.14 ± 0.00 | 0.00 | <1 |
| Benzoic acid | 879.10 | 121.03 | 0.05 ± 0.00 |  | 0.05 ± 0.00 | 0.90 | <1 |  | 0.07 ± 0.00 | > 0.05 | <1 |  | 0.08 ± 0.01 | > 0.05 | <1 |
| Phenylacetic acid | 842.10 | 135.04 | 0.05 ± 0.01 |  | 0.06 ± 0.00 | 0.57 | <1 |  | 0.06 ± 0.01 | > 0.05 | <1 |  | 0.09 ± 0.00 | 0.02 | <1 |
| Uracil | 690.67 | 111.02 | 0.05 ± 0.00 |  | 0.01 ± 0.00 | 0.20 | 1.23 |  | 0.04 ± 0.01 | > 0.05 | <1 |  | 0.02 ± 0.00 | > 0.05 | 1.16 |
| Pyrrole-2-carboxylic acid | 856.87 | 110.02 | 0.05 ± 0.01 |  | 0.02 ± 0.00 | 0.75 | 1.01 |  | 0.14 ± 0.00 | > 0.05 | <1 |  | 0.05 ± 0.00 | 0.01 | 1.08 |
| Ethyl tetradecanoate | 669.79 | 255.23 | 0.05 ± 0.00 |  | 0.02 ± 0.00 | 0.00 | 1.24 |  | 0.03 ± 0.00 | 0.00 | 1.24 |  | 0.02 ± 0.00 | > 0.05 | 1.04 |
| 3,4-Dihydroxyphenylglycol | 625.72 | 169.05 | 0.05 ± 0.00 |  | 0.05 ± 0.00 | 0.39 | <1 |  | 0.02 ± 0.00 | > 0.05 | <1 |  | 0.10 ± 0.01 | > 0.05 | <1 |
| Citicoline | 347.26 | 487.10 | 0.05 ± 0.01 |  | 0.02 ± 0.00 | 0.13 | 1.07 |  | 0.04 ± 0.00 | > 0.05 | <1 |  | 0.05 ± 0.00 | > 0.05 | 1.09 |
| Trehalose | 149.72 | 341.11 | 0.05 ± 0.00 |  | 0.07 ± 0.00 | 0.07 | 1.14 |  | 0.09 ± 0.00 | 0.00 | 1.22 |  | 0.16 ± 0.03 | > 0.05 | <1 |
| Gluconolactone | 43.94 | 177.04 | 0.05 ± 0.00 |  | 0.00 ± 0.00 | 0.03 | 1.24 |  | 0.02 ± 0.00 | 0.01 | 1.17 |  | 0.05 ± 0.00 | 0.01 | <1 |
| Pimelic acid | 623.12 | 159.07 | 0.04 ± 0.00 |  | 0.05 ± 0.00 | 0.57 | <1 |  | 0.08 ± 0.01 | > 0.05 | <1 |  | 0.04 ± 0.00 | > 0.05 | 1.15 |
| Pantothenic acid | 191.46 | 218.10 | 0.04 ± 0.00 |  | 0.02 ± 0.00 | 0.00 | 1.24 |  | 0.01 ± 0.00 | 0.00 | 1.24 |  | 0.01 ± 0.00 | 0.00 | <1 |
| Malic acid | 357.38 | 133.01 | 0.04 ± 0.00 |  | 0.05 ± 0.00 | 0.92 | <1 |  | 0.04 ± 0.01 | 0.01 | <1 |  | 0.10 ± 0.00 | > 0.05 | 1.11 |
| Dopamine | 234.78 | 299.08 | 0.04 ± 0.01 |  | 0.06 ± 0.01 | 0.00 | <1 |  | 0.04 ± 0.00 | 0.01 | 1.15 |  | 0.10 ± 0.02 | 0.02 | 1.12 |
| Erythrono-1,4-lactone | 153.27 | 117.02 | 0.04 ± 0.00 |  | 0.04 ± 0.00 | 0.22 | <1 |  | 0.04 ± 0.01 | 0.05 | 1.03 |  | 0.04 ± 0.01 | 0.00 | <1 |
| [4]-Gingerdiol 3,5-diacetate | 464.74 | 351.18 | 0.04 ± 0.00 |  | 0.02 ± 0.00 | 0.09 | 1.21 |  | 0.04 ± 0.00 | > 0.05 | <1 |  | 0.03 ± 0.00 | > 0.05 | <1 |
| Dihydrolipoate | 44.49 | 207.05 | 0.04 ± 0.00 |  | 0.03 ± 0.00 | 0.15 | 1.23 |  | 0.02 ± 0.00 | > 0.05 | <1 |  | 0.02 ± 0.00 | > 0.05 | <1 |
| α-Ketoisovaleric acid | 91.22 | 115.04 | 0.04 ± 0.01 |  | 0.01 ± 0.00 | 0.00 | 1.08 |  | 0.03 ± 0.00 | 0.00 | 1.19 |  | 0.01 ± 0.00 | > 0.05 | 1.14 |
| Docosahexaenoic acid | 373.87 | 327.23 | 0.04 ± 0.00 |  | 0.02 ± 0.00 | 0.02 | 1.23 |  | 0.04 ± 0.00 | > 0.05 | <1 |  | 0.01 ± 0.00 | 0.03 | 1.16 |
| But-2-enoic acid | 50.55 | 85.03 | 0.04 ± 0.00 |  | 0.01 ± 0.00 | 0.10 | 1.23 |  | 0.03 ± 0.00 | 0.00 | 1.23 |  | 0.02 ± 0.00 | 0.01 | 1.14 |
| 3,4-Dihydroxy-trans-cinnamate | 246.51 | 179.03 | 0.04 ± 0.01 |  | 0.01 ± 0.00 | 0.00 | 1.15 |  | 0.07 ± 0.00 | 0.00 | 1.26 |  | 0.05 ± 0.00 | 0.00 | 1.15 |
| Gingerol | 440.60 | 293.18 | 0.04 ± 0.00 |  | 0.11 ± 0.00 | 0.00 | 1.24 |  | 0.20 ± 0.01 | 0.02 | 1.12 |  | 0.09 ± 0.02 | 0.00 | 1.11 |
| Allocystathionine | 698.00 | 221.06 | 0.04 ± 0.00 |  | 0.02 ± 0.00 | 0.56 | 1.24 |  | 0.03 ± 0.00 | > 0.05 | <1 |  | 0.04 ± 0.00 | 0.00 | 1.16 |
| Linoleic acid | 502.59 | 279.23 | 0.04 ± 0.00 |  | 0.03 ± 0.00 | 0.00 | 1.22 |  | 0.03 ± 0.00 | 0.00 | 1.26 |  | 0.02 ± 0.00 | 0.02 | 1.15 |
| Theaflavin | 284.97 | 563.12 | 0.04 ± 0.00 |  | 0.04 ± 0.00 | 0.01 | <1 |  | 0.01 ± 0.00 | > 0.05 | <1 |  | 0.11 ± 0.01 | > 0.05 | 1.13 |
| Acetylisoniazid | 780.31 | 178.06 | 0.04 ± 0.00 |  | 0.06 ± 0.00 | 0.61 | 1.21 |  | 0.17 ± 0.01 | > 0.05 | <1 |  | 0.08 ± 0.00 | 0.00 | 1.15 |
| Cortisol | 526.69 | 361.20 | 0.04 ± 0.00 |  | 0.01 ± 0.00 | 0.00 | 1.24 |  | 0.02 ± 0.00 | 0.04 | 1.04 |  | 0.01 ± 0.00 | 0.00 | 1.13 |
| Glycitein | 299.00 | 283.06 | 0.04 ± 0.00 |  | 0.01 ± 0.00 | 0.00 | 1.18 |  | 0.05 ± 0.00 | 0.04 | <1 |  | 0.02 ± 0.00 | 0.00 | 1.14 |
| Succinic acid semialdehyde | 23.08 | 101.02 | 0.04 ± 0.01 |  | 0.12 ± 0.00 | 0.28 | 1.23 |  | 0.30 ± 0.01 | > 0.05 | <1 |  | 0.12 ± 0.01 | > 0.05 | 1.15 |
| Leukotriene B4 | 413.87 | 335.22 | 0.04 ± 0.00 |  | 0.00 ± 0.00 | 0.02 | 1.24 |  | 0.04 ± 0.00 | > 0.05 | <1 |  | 0.01 ± 0.00 | 0.03 | 1.14 |
| Guanosine | 187.48 | 282.08 | 0.04 ± 0.00 |  | 0.01 ± 0.00 | 0.00 | 1.16 |  | 0.01 ± 0.00 | 0.00 | 1.26 |  | 0.02 ± 0.00 | 0.00 | 1.16 |
| Phenylglyoxylic acid | 786.12 | 149.02 | 0.03 ± 0.01 |  | 0.01 ± 0.00 | 0.42 | <1 |  | 0.04 ± 0.00 | > 0.05 | <1 |  | 0.02 ± 0.00 | 0.01 | <1 |
| (2R)-6,8-Diglucopyranosyl-4',5,7-trihydroxyflavanone | 85.07 | 595.17 | 0.03 ± 0.00 |  | 0.04 ± 0.00 | 0.36 | <1 |  | 0.03 ± 0.00 | 0.01 | 1.18 |  | 0.07 ± 0.00 | 0.05 | 1.06 |
| D-Tartaric acid | 71.86 | 149.01 | 0.03 ± 0.00 |  | 0.03 ± 0.00 | 0.36 | 1.19 |  | 0.11 ± 0.00 | > 0.05 | <1 |  | 0.06 ± 0.00 | 0.01 | 1.15 |
| 6β-Hydroxytestosterone | 500.50 | 303.20 | 0.03 ± 0.00 |  | 0.01 ± 0.00 | 0.00 | 1.23 |  | 0.03 ± 0.00 | > 0.05 | <1 |  | 0.02 ± 0.00 | 0.00 | 1.12 |
| Epiandrosterone | 640.46 | 289.22 | 0.03 ± 0.00 |  | 0.06 ± 0.00 | 0.00 | 1.17 |  | 0.04 ± 0.00 | 0.01 | 1.16 |  | 0.06 ± 0.00 | 0.00 | 1.11 |
| 21-Hydroxypregnenolone | 605.32 | 331.23 | 0.03 ± 0.00 |  | 0.03 ± 0.00 | 0.00 | <1 |  | 0.04 ± 0.00 | 0.00 | 1.24 |  | 0.07 ± 0.00 | 0.00 | 1.15 |
| Myristoleic acid | 589.86 | 225.19 | 0.03 ± 0.00 |  | 0.04 ± 0.01 | 0.47 | <1 |  | 0.04 ± 0.00 | 0.01 | 1.16 |  | 0.07 ± 0.01 | 0.01 | 1.16 |
| Dehydroepiandrosterone | 547.48 | 287.20 | 0.03 ± 0.00 |  | 0.02 ± 0.00 | 0.00 | 1.21 |  | 0.04 ± 0.00 | 0.00 | 1.21 |  | 0.01 ± 0.00 | 0.00 | 1.10 |
| Glycyrrhetinic acid | 620.05 | 469.33 | 0.03 ± 0.01 |  | 0.00 ± 0.00 | 0.00 | <1 |  | 0.04 ± 0.00 | 0.00 | 1.26 |  | 0.00 ± 0.00 | 0.00 | 1.01 |
| Formononetin | 430.06 | 267.07 | 0.03 ± 0.00 |  | 0.03 ± 0.00 | 0.00 | <1 |  | 0.03 ± 0.00 | 0.00 | 1.22 |  | 0.03 ± 0.00 | 0.00 | 1.15 |
| Uridine | 52.59 | 243.06 | 0.03 ± 0.00 |  | 0.01 ± 0.00 | 0.07 | 1.22 |  | 0.02 ± 0.00 | 0.00 | 1.22 |  | 0.00 ± 0.00 | 0.00 | 1.16 |
| all-trans-Retinoic acid | 567.86 | 299.20 | 0.03 ± 0.00 |  | 0.02 ± 0.00 | 0.00 | 1.17 |  | 0.07 ± 0.00 | > 0.05 | <1 |  | 0.04 ± 0.00 | > 0.05 | 1.16 |
| Acetaminophen | 85.71 | 150.06 | 0.03 ± 0.00 |  | 0.00 ± 0.00 | 0.00 | 1.22 |  | 0.01 ± 0.00 | 0.00 | 1.26 |  | 0.02 ± 0.00 | > 0.05 | <1 |
| (R)-Kawain | 274.17 | 229.09 | 0.03 ± 0.00 |  | 0.01 ± 0.00 | 0.00 | 1.22 |  | 0.03 ± 0.00 | 0.00 | <1 |  | 0.00 ± 0.00 | > 0.05 | <1 |
| benzene-1,2,4-triol | 43.93 | 125.04 | 0.03 ± 0.00 |  | 0.01 ± 0.00 | 0.27 | 1.20 |  | 0.02 ± 0.00 | > 0.05 | <1 |  | 0.02 ± 0.00 | > 0.05 | 1.16 |
| D-Glucurono-6,3-lactone | 47.30 | 175.02 | 0.03 ± 0.00 |  | 0.01 ± 0.00 | 0.01 | 1.13 |  | 0.02 ± 0.02 | 0.00 | 1.20 |  | 0.01 ± 0.00 | 0.00 | 1.16 |
| 2-Isopropylmalic acid | 665.28 | 175.06 | 0.03 ± 0.00 |  | 0.02 ± 0.00 | 0.41 | 1.17 |  | 0.02 ± 0.00 | > 0.05 | <1 |  | 0.03 ± 0.00 | > 0.05 | 1.14 |
| Chorismate | 225.24 | 225.04 | 0.03 ± 0.01 |  | 0.03 ± 0.00 | 0.00 | <1 |  | 0.03 ± 0.00 | 0.00 | 1.26 |  | 0.04 ± 0.00 | 0.00 | <1 |
| 6β-Hydroxyasiatic acid | 414.67 | 503.34 | 0.03 ± 0.00 |  | 0.01 ± 0.00 | 0.00 | 1.20 |  | 0.02 ± 0.00 | 0.00 | 1.26 |  | 0.04 ± 0.00 | 0.00 | <1 |
| N1-(2-Hydroxyethyl)flurazepam | 192.89 | 331.07 | 0.02 ± 0.00 |  | 0.01 ± 0.00 | 0.00 | 1.22 |  | 0.03 ± 0.00 | 0.00 | 1.23 |  | 0.01 ± 0.00 | 0.03 | 1.13 |
| Salicyluric acid | 678.83 | 194.05 | 0.02 ± 0.00 |  | 0.03 ± 0.01 | > 0.05 | <1 |  | 0.27 ± 0.05 | 0.02 | 1.12 |  | 0.07 ± 0.02 | 0.01 | 1.16 |
| L-Gulonolactone | 73.46 | 177.04 | 0.02 ± 0.00 |  | 0.02 ± 0.00 | > 0.05 | <1 |  | 0.04 ± 0.01 | 0.02 | 1.13 |  | 0.07 ± 0.04 | 0.00 | 1.16 |
| 6-Hydroxynicotinic acid | 820.55 | 138.02 | 0.02 ± 0.00 |  | 0.01 ± 0.00 | > 0.05 | 1.23 |  | 0.01 ± 0.00 | 0.01 | 1.18 |  | 0.02 ± 0.00 | > 0.05 | 1.16 |
| 2-Methoxyestrone | 558.85 | 299.17 | 0.02 ± 0.00 |  | 0.04 ± 0.00 | 0.00 | 1.19 |  | 0.04 ± 0.00 | > 0.05 | <1 |  | 0.04 ± 0.00 | > 0.05 | 1.15 |
| Stearic acid | 793.67 | 283.26 | 0.02 ± 0.00 |  | 0.00 ± 0.00 | > 0.05 | 1.22 |  | 0.03 ± 0.00 | 0.00 | 1.23 |  | 0.04 ± 0.00 | 0.00 | <1 |
| Gallic acid | 784.86 | 169.01 | 0.02 ± 0.00 |  | 0.05 ± 0.00 | > 0.05 | 1.24 |  | 0.06 ± 0.00 | 0.00 | 1.25 |  | 0.04 ± 0.00 | 0.00 | 1.11 |
| Traumatic acid | 351.89 | 227.13 | 0.02 ± 0.00 |  | 0.02 ± 0.00 | > 0.05 | <1 |  | 0.01 ± 0.00 | > 0.05 | <1 |  | 0.02 ± 0.00 | 0.02 | 1.16 |
| Geranylacetone | 509.24 | 329.29 | 0.02 ± 0.00 |  | 0.02 ± 0.00 | 0.03 | <1 |  | 0.02 ± 0.00 | 0.02 | 1.09 |  | 0.04 ± 0.00 | > 0.05 | 1.13 |
| 8-iso-15-keto-PGE2 | 407.42 | 349.20 | 0.02 ± 0.01 |  | 0.01 ± 0.00 | 0.00 | <1 |  | 0.00 ± 0.00 | 0.00 | 1.25 |  | 0.01 ± 0.00 | 0.00 | 1.15 |
| 4-hydroxy-3-[1-(4-hydroxyphenyl)-3-oxobutyl]-2H-chromen-2-one | 445.59 | 323.09 | 0.02 ± 0.00 |  | 0.02 ± 0.00 | 0.00 | <1 |  | 0.03 ± 0.00 | 0.00 | 1.24 |  | 0.07 ± 0.00 | 0.00 | 1.16 |
| [8]-Dehydrogingerdione | 433.40 | 317.18 | 0.02 ± 0.00 |  | 0.04 ± 0.00 | 0.00 | 1.24 |  | 0.02 ± 0.01 | > 0.05 | <1 |  | 0.02 ± 0.01 | > 0.05 | 1.12 |
| 5,7-dihydroxy-2-(4-hydroxy-3,5-dimethoxyphenyl)-4H-chromen-4-one | 348.42 | 329.07 | 0.02 ± 0.00 |  | 0.06 ± 0.00 | > 0.05 | 1.23 |  | 0.03 ± 0.00 | 0.02 | 1.12 |  | 0.04 ± 0.00 | > 0.05 | 1.16 |
| (10E,12Z)-9-HODE | 534.73 | 295.23 | 0.02 ± 0.01 |  | 0.01 ± 0.00 | 0.00 | <1 |  | 0.01 ± 0.00 | 0.00 | 1.26 |  | 0.00 ± 0.00 | 0.00 | <1 |
| 3-Hydroxycapric acid | 374.54 | 187.13 | 0.02 ± 0.00 |  | 0.00 ± 0.00 | > 0.05 | 1.20 |  | 0.02 ± 0.00 | > 0.05 | <1 |  | 0.01 ± 0.00 | 0.00 | <1 |
| myo-Inositol | 43.82 | 179.06 | 0.02 ± 0.00 |  | 0.00 ± 0.00 | 0.00 | 1.23 |  | 0.01 ± 0.00 | 0.00 | 1.26 |  | 0.01 ± 0.00 | 0.00 | 1.16 |
| Mannitol | 54.09 | 181.07 | 0.01 ± 0.00 |  | 0.01 ± 0.00 | > 0.05 | 1.16 |  | 0.03 ± 0.00 | 0.00 | 1.22 |  | 0.01 ± 0.00 | 0.01 | 1.16 |
| Glycitin | 345.74 | 445.11 | 0.01 ± 0.00 |  | 0.01 ± 0.00 | > 0.05 | <1 |  | 0.08 ± 0.01 | > 0.05 | <1 |  | 0.01 ± 0.00 | 0.00 | 1.15 |
| Methyldopa | 769.10 | 210.08 | 0.01 ± 0.00 |  | 0.02 ± 0.00 | > 0.05 | 1.11 |  | 0.01 ± 0.00 | > 0.05 | <1 |  | 0.03 ± 0.00 | > 0.05 | 1.15 |
| Eudesmic acid | 294.08 | 211.06 | 0.01 ± 0.00 |  | 0.01 ± 0.00 | 0.00 | <1 |  | 0.02 ± 0.00 | 0.00 | 1.25 |  | 0.02 ± 0.00 | 0.00 | 1.14 |
| trans-Piceid | 335.45 | 403.14 | 0.01 ± 0.00 |  | 0.02 ± 0.00 | > 0.05 | 1.19 |  | 0.02 ± 0.00 | > 0.05 | <1 |  | 0.02 ± 0.00 | 0.00 | 1.16 |
| 15-KETE | 609.84 | 317.21 | 0.01 ± 0.01 |  | 0.00 ± 0.00 | 0.00 | <1 |  | 0.02 ± 0.00 | 0.00 | 1.26 |  | 0.00 ± 0.00 | 0.00 | 1.16 |
| LysoPE(18:1(9Z)/0:0) | 549.75 | 478.29 | 0.01 ± 0.00 |  | 0.01 ± 0.00 | > 0.05 | <1 |  | 0.01 ± 0.00 | > 0.05 | 1.01 |  | 0.02 ± 0.00 | > 0.05 | 1.16 |
| 5-Methoxysalicylic acid | 284.86 | 167.03 | 0.01 ± 0.00 |  | 0.01 ± 0.00 | 0.00 | <1 |  | 0.03 ± 0.00 | 0.00 | 1.25 |  | 0.02 ± 0.00 | 0.00 | <1 |
| 3-(3,4-Dihydroxy-5-methoxy)-2-propenoic acid | 232.60 | 209.05 | 0.01 ± 0.00 |  | 0.01 ± 0.00 | 0.00 | <1 |  | 0.01 ± 0.00 | > 0.05 | <1 |  | 0.01 ± 0.00 | 0.00 | 1.16 |
| Byssochlamic acid | 311.40 | 331.12 | 0.01 ± 0.00 |  | 0.09 ± 0.08 | 0.01 | <1 |  | 0.02 ± 0.00 | > 0.05 | <1 |  | 0.02 ± 0.00 | 0.04 | 1.15 |
| Raffinose | 75.10 | 503.16 | 0.01 ± 0.00 |  | 0.01 ± 0.00 | 0.00 | <1 |  | 0.00 ± 0.00 | > 0.05 | <1 |  | 0.01 ± 0.00 | > 0.05 | 1.14 |
| Glycyltyrosine | 651.68 | 237.09 | 0.00 ± 0.00 |  | 0.00 ± 0.00 | > 0.05 | <1 |  | 0.00 ± 0.00 | > 0.05 | <1 |  | 0.00 ± 0.00 | > 0.05 | 1.14 |
| Gibberellin A19 | 361.17 | 361.17 | 0.00 ± 0.00 |  | 0.01 ± 0.00 | 0.00 | 1.13 |  | 0.00 ± 0.00 | 0.00 | 1.21 |  | 0.00 ± 0.00 | 0.00 | <1 |
| (R)-3-Hydroxy-tetradecanoic acid | 458.69 | 243.20 | 0.00 ± 0.00 |  | 0.01 ± 0.00 | 0.03 | 1.22 |  | 0.01 ± 0.00 | 0.04 | 1.05 |  | 0.01 ± 0.00 | 0.04 | 1.15 |
| Carnosic acid | 590.60 | 331.19 | 0.00 ± 0.00 |  | 0.00 ± 0.00 | 0.00 | <1 |  | 0.00 ± 0.00 | 0.02 | 1.13 |  | 0.01 ± 0.00 | 0.00 | 1.16 |
| 5-Hydroxy-L-tryptophan | 223.61 | 219.08 | 0.00 ± 0.00 |  | 0.00 ± 0.00 | 0.00 | <1 |  | 0.00 ± 0.00 | 0.00 | 1.25 |  | 0.01 ± 0.00 | 0.00 | 1.16 |
| Ascochitine | 340.38 | 275.09 | 0.00 ± 0.00 |  | 0.00 ± 0.00 | 0.00 | 1.15 |  | 0.01 ± 0.00 | > 0.05 | <1 |  | 0.01 ± 0.00 | 0.00 | 1.13 |

CK, T1, T2 and T3 represent roots in the control, 25 mg kg^-1^ Cd, 50 mg kg^-1^ Cd, and 100 mg kg^-1^ Cd treated groups (n = 3), respectively (the same below). All data are presented as the mean ± SE.
