# Supplementary material for: Untargeted LC–MS/MS-Based Metabolomic Profiling for the Edible and Medicinal Plant Salvia miltiorrhiza Under Different Levels of Cadmium Stress
Source: Front Plant Sci. 2022 Jul 28;13:889370. doi: 10.3389/fpls.2022.889370 (PMC9366474; doi:10.3389/fpls.2022.889370)
Supplement: Supplementary Table S3 — Relative contents of metabolites of different types in S. miltiorrhiza roots with different levels of Cd stress. CK, T1, T2 and T3 represent roots in the control, 25 mg kg−1 Cd, 50 mg kg−1 Cd, and 100 mg kg−1 Cd treated groups, respectively (the same below). Different lowercase letters indicate significant differences within each treatment in the roots (p < 0.05). All data are presented as the mean ± SE (n = 3). [file Table_3.DOCX]

Supply table 3 Relative contents of metabolites of different types in S. miltiorrhiza roots with different levels of Cd stress.

| Metabolites | CK | T1 | T2 | T3 |
| --- | --- | --- | --- | --- |
| Amino acids | 47.83 ± 1.08 a | 57.70 ± 1.26 b | 89.20 ± 3.99 c | 92.80 ± 7.36 c |
| Organic acids | 352.51 ± 37.27 ab | 285.54 ± 26.97 a | 333.02 ± 30.11 a | 579.49 ± 84.83 b |
| Fatty acids | 139.90 ± 1.56 a | 17.98 ± 0.20 b | 57.55 ± 1.03 c | 35.56 ± 0.59 d |
| Ketones | 39.84 ± 0.74 a | 21.40 ± 2.00 b | 35.28 ± 1.86 a | 40.96 ± 6.09 a |
| Sugars | 46.18 ± 2.59 a | 60.33 ± 1.58 b | 41.84 ± 1.73 ac | 33.76 ± 3.55 c |
| Amides | 58.63 ± 0.43 a | 31.50 ± 0.28 b | 51.55 ± 0.84 c | 73.97 ± 1.30 d |
| Others | 58.80 ± 0.45 a | 33.78 ± 0.33 b | 55.30 ± 0.39 c | 90.99 ± 2.27 d |

CK, T1, T2 and T3 represent roots in the control, 25 mg kg^-1^ Cd, 50 mg kg^-1^ Cd, and 100 mg kg^-1^ Cd treated groups, respectively (the same below). Different lowercase letters indicate significant differences within each treatment in the roots (*p* < 0.05). All data are presented as the mean ± SE (n=3).
