# Supplementary material for: Untargeted LC–MS/MS-Based Metabolomic Profiling for the Edible and Medicinal Plant Salvia miltiorrhiza Under Different Levels of Cadmium Stress
Source: Front Plant Sci. 2022 Jul 28;13:889370. doi: 10.3389/fpls.2022.889370 (PMC9366474; doi:10.3389/fpls.2022.889370)
Supplement: Supplementary Table S4 — Results of pathway analysis involving all of the discriminating metabolites in S. miltiorrhiza roots with different levels of Cd stress. All pathways shown in the table are potential target metabolic pathways with pathway impacts above 0.1; Total Cmpd, total number of compounds in the pathway; Hits, the number of actually matched compounds in the pathway; Holm adjust, p value adjusted by the Holm–Bonferroni method; FDR, p value adjusted using the False Discovery Rate; Impact, pathway impact value. [file Table_4.DOCX]

Supply table 4 Results of pathway analysis involving all of the discriminating metabolites in *S. miltiorrhiza* roots with different levels of Cd stress.

| Groups | Pathway | Total | Expected | Hits | Raw p | -LOG10(p) | Holm adjust | FDR | Impact |
| --- | --- | --- | --- | --- | --- | --- | --- | --- | --- |
| T1 | Biosynthesis of unsaturated fatty acids | 22 | 1.83 | 7 | 0.00 | 2.86 | 0.13 | 0.13 | 0.00 |
|  | Isoquinoline alkaloid biosynthesis | 6 | 0.50 | 3 | 0.01 | 2.03 | 0.88 | 0.45 | 1.00 |
|  | Betalain biosynthesis | 3 | 0.25 | 2 | 0.02 | 1.71 | 1.00 | 0.62 | 1.00 |
|  | Aminoacyl-tRNA biosynthesis | 46 | 3.84 | 8 | 0.03 | 1.49 | 1.00 | 0.77 | 0.11 |
|  | Alanine, aspartate and glutamate metabolism | 22 | 1.83 | 4 | 0.10 | 0.98 | 1.00 | 1.00 | 0.26 |
|  | beta-Alanine metabolism | 18 | 1.50 | 3 | 0.18 | 0.73 | 1.00 | 1.00 | 0.25 |
|  | Tyrosine metabolism | 18 | 1.50 | 3 | 0.18 | 0.73 | 1.00 | 1.00 | 0.28 |
|  | Glyoxylate and dicarboxylate metabolism | 29 | 2.42 | 4 | 0.22 | 0.66 | 1.00 | 1.00 | 0.08 |
|  | Citrate cycle (TCA cycle) | 20 | 1.67 | 3 | 0.23 | 0.64 | 1.00 | 1.00 | 0.19 |
|  | Arachidonic acid metabolism | 12 | 1.00 | 2 | 0.26 | 0.58 | 1.00 | 1.00 | 0.00 |
|  | Phenylalanine, tyrosine and tryptophan biosynthesis | 22 | 1.83 | 3 | 0.28 | 0.56 | 1.00 | 1.00 | 0.17 |
|  | Linoleic acid metabolism | 4 | 0.33 | 1 | 0.29 | 0.53 | 1.00 | 1.00 | 1.00 |
|  | Pantothenate and CoA biosynthesis | 23 | 1.92 | 3 | 0.30 | 0.52 | 1.00 | 1.00 | 0.23 |
|  | Cutin, suberine and wax biosynthesis | 14 | 1.17 | 2 | 0.33 | 0.48 | 1.00 | 1.00 | 0.25 |
|  | Sulfur metabolism | 15 | 1.25 | 2 | 0.36 | 0.44 | 1.00 | 1.00 | 0.03 |
|  | Cyanoamino acid metabolism | 26 | 2.17 | 3 | 0.37 | 0.43 | 1.00 | 1.00 | 0.00 |
|  | Galactose metabolism | 27 | 2.25 | 3 | 0.39 | 0.40 | 1.00 | 1.00 | 0.09 |
|  | Biosynthesis of secondary metabolites - other antibiotics | 6 | 0.50 | 1 | 0.41 | 0.39 | 1.00 | 1.00 | 0.00 |
|  | Arginine and proline metabolism | 28 | 2.34 | 3 | 0.42 | 0.38 | 1.00 | 1.00 | 0.14 |
|  | Histidine metabolism | 17 | 1.42 | 2 | 0.42 | 0.38 | 1.00 | 1.00 | 0.04 |
|  | Butanoate metabolism | 17 | 1.42 | 2 | 0.42 | 0.38 | 1.00 | 1.00 | 0.14 |
|  | Ascorbate and aldarate metabolism | 18 | 1.50 | 2 | 0.45 | 0.35 | 1.00 | 1.00 | 0.00 |
|  | Arginine biosynthesis | 18 | 1.50 | 2 | 0.45 | 0.35 | 1.00 | 1.00 | 0.08 |
|  | Monobactam biosynthesis | 8 | 0.67 | 1 | 0.50 | 0.30 | 1.00 | 1.00 | 0.00 |
|  | Tropane, piperidine and pyridine alkaloid biosynthesis | 8 | 0.67 | 1 | 0.50 | 0.30 | 1.00 | 1.00 | 0.00 |
|  | Carbon fixation in photosynthetic organisms | 21 | 1.75 | 2 | 0.53 | 0.27 | 1.00 | 1.00 | 0.06 |
|  | Lysine biosynthesis | 9 | 0.75 | 1 | 0.54 | 0.26 | 1.00 | 1.00 | 0.00 |
|  | Valine, leucine and isoleucine biosynthesis | 22 | 1.83 | 2 | 0.56 | 0.25 | 1.00 | 1.00 | 0.11 |
|  | Ubiquinone and other terpenoid-quinone biosynthesis | 35 | 2.92 | 3 | 0.57 | 0.24 | 1.00 | 1.00 | 0.00 |
|  | Phenylpropanoid biosynthesis | 35 | 2.92 | 3 | 0.57 | 0.24 | 1.00 | 1.00 | 0.03 |
|  | Caffeine metabolism | 10 | 0.83 | 1 | 0.58 | 0.23 | 1.00 | 1.00 | 0.00 |
|  | Tryptophan metabolism | 23 | 1.92 | 2 | 0.58 | 0.23 | 1.00 | 1.00 | 0.00 |
|  | Stilbenoid, diarylheptanoid and gingerol biosynthesis | 11 | 0.92 | 1 | 0.62 | 0.21 | 1.00 | 1.00 | 0.00 |
|  | Vitamin B6 metabolism | 11 | 0.92 | 1 | 0.62 | 0.21 | 1.00 | 1.00 | 0.21 |
|  | Phenylalanine metabolism | 12 | 1.00 | 1 | 0.65 | 0.19 | 1.00 | 1.00 | 0.42 |
|  | Nicotinate and nicotinamide metabolism | 13 | 1.08 | 1 | 0.68 | 0.17 | 1.00 | 1.00 | 0.00 |
|  | Inositol phosphate metabolism | 28 | 2.34 | 2 | 0.69 | 0.16 | 1.00 | 1.00 | 0.10 |
|  | Fatty acid biosynthesis | 56 | 4.67 | 4 | 0.70 | 0.15 | 1.00 | 1.00 | 0.01 |
|  | Diterpenoid biosynthesis | 47 | 3.92 | 3 | 0.77 | 0.11 | 1.00 | 1.00 | 0.06 |
|  | Sphingolipid metabolism | 17 | 1.42 | 1 | 0.77 | 0.11 | 1.00 | 1.00 | 0.00 |
|  | Glycine, serine and threonine metabolism | 33 | 2.75 | 2 | 0.78 | 0.11 | 1.00 | 1.00 | 0.18 |
|  | Pentose phosphate pathway | 19 | 1.58 | 1 | 0.81 | 0.09 | 1.00 | 1.00 | 0.00 |
|  | Propanoate metabolism | 20 | 1.67 | 1 | 0.83 | 0.08 | 1.00 | 1.00 | 0.00 |
|  | Valine, leucine and isoleucine degradation | 37 | 3.09 | 2 | 0.83 | 0.08 | 1.00 | 1.00 | 0.02 |
|  | Starch and sucrose metabolism | 22 | 1.83 | 1 | 0.85 | 0.07 | 1.00 | 1.00 | 0.14 |
|  | Pyruvate metabolism | 22 | 1.83 | 1 | 0.85 | 0.07 | 1.00 | 1.00 | 0.15 |
|  | Fatty acid elongation | 23 | 1.92 | 1 | 0.87 | 0.06 | 1.00 | 1.00 | 0.00 |
|  | Phosphatidylinositol signaling system | 26 | 2.17 | 1 | 0.90 | 0.05 | 1.00 | 1.00 | 0.03 |
|  | Folate biosynthesis | 27 | 2.25 | 1 | 0.91 | 0.04 | 1.00 | 1.00 | 0.00 |
|  | Glutathione metabolism | 27 | 2.25 | 1 | 0.91 | 0.04 | 1.00 | 1.00 | 0.05 |
|  | alpha-Linolenic acid metabolism | 27 | 2.25 | 1 | 0.91 | 0.04 | 1.00 | 1.00 | 0.11 |
|  | Cysteine and methionine metabolism | 46 | 3.84 | 2 | 0.91 | 0.04 | 1.00 | 1.00 | 0.00 |
|  | Terpenoid backbone biosynthesis | 29 | 2.42 | 1 | 0.92 | 0.04 | 1.00 | 1.00 | 0.03 |
|  | Fatty acid degradation | 37 | 3.09 | 1 | 0.96 | 0.02 | 1.00 | 1.00 | 0.00 |
|  | Pyrimidine metabolism | 38 | 3.17 | 1 | 0.97 | 0.02 | 1.00 | 1.00 | 0.00 |
|  | Carotenoid biosynthesis | 42 | 3.50 | 1 | 0.98 | 0.01 | 1.00 | 1.00 | 0.00 |
|  | Purine metabolism | 63 | 5.25 | 1 | 1.00 | 0.00 | 1.00 | 1.00 | 0.00 |
| T2 | Isoquinoline alkaloid biosynthesis | 6 | 0.46 | 3 | 0.01 | 2.13 | 0.72 | 0.54 | 1.00 |
|  | Betalain biosynthesis | 3 | 0.23 | 2 | 0.02 | 1.78 | 1.00 | 0.54 | 1.00 |
|  | Aminoacyl-tRNA biosynthesis | 46 | 3.54 | 8 | 0.02 | 1.68 | 1.00 | 0.54 | 0.11 |
|  | Biosynthesis of unsaturated fatty acids | 22 | 1.69 | 5 | 0.02 | 1.65 | 1.00 | 0.54 | 0.00 |
|  | Tyrosine metabolism | 16 | 1.23 | 4 | 0.03 | 1.53 | 1.00 | 0.56 | 0.11 |
|  | C5-Branched dibasic acid metabolism | 6 | 0.46 | 2 | 0.07 | 1.14 | 1.00 | 0.99 | 0.00 |
|  | Alanine, aspartate and glutamate metabolism | 22 | 1.69 | 4 | 0.08 | 1.08 | 1.00 | 0.99 | 0.13 |
|  | Valine, leucine and isoleucine biosynthesis | 22 | 1.69 | 4 | 0.08 | 1.08 | 1.00 | 0.99 | 0.29 |
|  | Pantothenate and CoA biosynthesis | 23 | 1.77 | 4 | 0.09 | 1.03 | 1.00 | 1.00 | 0.23 |
|  | Monobactam biosynthesis | 8 | 0.61 | 2 | 0.12 | 0.92 | 1.00 | 1.00 | 0.00 |
|  | Ascorbate and aldarate metabolism | 18 | 1.38 | 3 | 0.16 | 0.81 | 1.00 | 1.00 | 0.00 |
|  | beta-Alanine metabolism | 18 | 1.38 | 3 | 0.16 | 0.81 | 1.00 | 1.00 | 0.25 |
|  | Cyanoamino acid metabolism | 29 | 2.23 | 4 | 0.18 | 0.75 | 1.00 | 1.00 | 0.00 |
|  | Citrate cycle (TCA cycle) | 20 | 1.54 | 3 | 0.19 | 0.71 | 1.00 | 1.00 | 0.07 |
|  | Carbon fixation in photosynthetic organisms | 21 | 1.61 | 3 | 0.22 | 0.67 | 1.00 | 1.00 | 0.09 |
|  | Phenylalanine, tyrosine and tryptophan biosynthesis | 22 | 1.69 | 3 | 0.24 | 0.63 | 1.00 | 1.00 | 0.17 |
|  | Glycine, serine and threonine metabolism | 33 | 2.54 | 4 | 0.24 | 0.61 | 1.00 | 1.00 | 0.18 |
|  | Linoleic acid metabolism | 4 | 0.31 | 1 | 0.27 | 0.56 | 1.00 | 1.00 | 1.00 |
|  | Sulfur metabolism | 15 | 1.15 | 2 | 0.32 | 0.49 | 1.00 | 1.00 | 0.03 |
|  | Histidine metabolism | 15 | 1.15 | 2 | 0.32 | 0.49 | 1.00 | 1.00 | 0.04 |
|  | Butanoate metabolism | 17 | 1.31 | 2 | 0.38 | 0.42 | 1.00 | 1.00 | 0.00 |
|  | Glyoxylate and dicarboxylate metabolism | 29 | 2.23 | 3 | 0.39 | 0.41 | 1.00 | 1.00 | 0.08 |
|  | Arginine biosynthesis | 18 | 1.38 | 2 | 0.41 | 0.39 | 1.00 | 1.00 | 0.08 |
|  | Cutin, suberine and wax biosynthesis | 18 | 1.38 | 2 | 0.41 | 0.39 | 1.00 | 1.00 | 0.13 |
|  | Propanoate metabolism | 20 | 1.54 | 2 | 0.46 | 0.33 | 1.00 | 1.00 | 0.03 |
|  | Tropane, piperidine and pyridine alkaloid biosynthesis | 8 | 0.61 | 1 | 0.47 | 0.32 | 1.00 | 1.00 | 0.00 |
|  | Cysteine and methionine metabolism | 46 | 3.54 | 4 | 0.48 | 0.32 | 1.00 | 1.00 | 0.08 |
|  | Starch and sucrose metabolism | 22 | 1.69 | 2 | 0.51 | 0.29 | 1.00 | 1.00 | 0.15 |
|  | Pyruvate metabolism | 22 | 1.69 | 2 | 0.51 | 0.29 | 1.00 | 1.00 | 0.32 |
|  | Lysine biosynthesis | 9 | 0.69 | 1 | 0.51 | 0.29 | 1.00 | 1.00 | 0.00 |
|  | Caffeine metabolism | 10 | 0.77 | 1 | 0.55 | 0.26 | 1.00 | 1.00 | 0.00 |
|  | Ubiquinone and other terpenoid-quinone biosynthesis | 38 | 2.92 | 3 | 0.57 | 0.24 | 1.00 | 1.00 | 0.00 |
|  | Phenylalanine metabolism | 11 | 0.85 | 1 | 0.59 | 0.23 | 1.00 | 1.00 | 0.47 |
|  | Galactose metabolism | 27 | 2.08 | 2 | 0.63 | 0.20 | 1.00 | 1.00 | 0.03 |
|  | Fatty acid biosynthesis | 56 | 4.30 | 4 | 0.64 | 0.20 | 1.00 | 1.00 | 0.01 |
|  | Tryptophan metabolism | 28 | 2.15 | 2 | 0.65 | 0.19 | 1.00 | 1.00 | 0.00 |
|  | Diterpenoid biosynthesis | 28 | 2.15 | 2 | 0.65 | 0.19 | 1.00 | 1.00 | 0.04 |
|  | Inositol phosphate metabolism | 28 | 2.15 | 2 | 0.65 | 0.19 | 1.00 | 1.00 | 0.10 |
|  | alpha-Linolenic acid metabolism | 28 | 2.15 | 2 | 0.65 | 0.19 | 1.00 | 1.00 | 0.11 |
|  | Nicotinate and nicotinamide metabolism | 13 | 1.00 | 1 | 0.65 | 0.19 | 1.00 | 1.00 | 0.00 |
|  | Terpenoid backbone biosynthesis | 30 | 2.31 | 2 | 0.69 | 0.16 | 1.00 | 1.00 | 0.03 |
|  | Pentose and glucuronate interconversions | 16 | 1.23 | 1 | 0.72 | 0.14 | 1.00 | 1.00 | 0.22 |
|  | Sphingolipid metabolism | 17 | 1.31 | 1 | 0.75 | 0.13 | 1.00 | 1.00 | 0.00 |
|  | Arginine and proline metabolism | 34 | 2.61 | 2 | 0.75 | 0.12 | 1.00 | 1.00 | 0.17 |
|  | Valine, leucine and isoleucine degradation | 37 | 2.84 | 2 | 0.79 | 0.10 | 1.00 | 1.00 | 0.02 |
|  | Pyrimidine metabolism | 38 | 2.92 | 2 | 0.80 | 0.09 | 1.00 | 1.00 | 0.03 |
|  | Thiamine metabolism | 22 | 1.69 | 1 | 0.83 | 0.08 | 1.00 | 1.00 | 0.00 |
|  | Fatty acid elongation | 23 | 1.77 | 1 | 0.84 | 0.07 | 1.00 | 1.00 | 0.00 |
|  | Phosphatidylinositol signaling system | 26 | 2.00 | 1 | 0.88 | 0.06 | 1.00 | 1.00 | 0.03 |
|  | Glutathione metabolism | 26 | 2.00 | 1 | 0.88 | 0.06 | 1.00 | 1.00 | 0.05 |
|  | Glycolysis / Gluconeogenesis | 26 | 2.00 | 1 | 0.88 | 0.06 | 1.00 | 1.00 | 0.12 |
|  | Folate biosynthesis | 27 | 2.08 | 1 | 0.89 | 0.05 | 1.00 | 1.00 | 0.00 |
|  | Glucosinolate biosynthesis | 65 | 5.00 | 3 | 0.89 | 0.05 | 1.00 | 1.00 | 0.00 |
|  | Fatty acid degradation | 37 | 2.84 | 1 | 0.95 | 0.02 | 1.00 | 1.00 | 0.00 |
|  | Purine metabolism | 63 | 4.84 | 2 | 0.96 | 0.02 | 1.00 | 1.00 | 0.00 |
|  | Carotenoid biosynthesis | 43 | 3.31 | 1 | 0.97 | 0.01 | 1.00 | 1.00 | 0.00 |
|  | Phenylpropanoid biosynthesis | 46 | 3.54 | 1 | 0.98 | 0.01 | 1.00 | 1.00 | 0.00 |
| T3 | Biosynthesis of unsaturated fatty acids | 22 | 1.99 | 5 | 0.04 | 1.38 | 1.00 | 1.00 | 0.00 |
|  | C5-Branched dibasic acid metabolism | 6 | 0.54 | 2 | 0.10 | 1.02 | 1.00 | 1.00 | 0.50 |
|  | Aminoacyl-tRNA biosynthesis | 46 | 4.16 | 7 | 0.11 | 0.94 | 1.00 | 1.00 | 0.11 |
|  | Alanine, aspartate and glutamate metabolism | 22 | 1.99 | 4 | 0.13 | 0.89 | 1.00 | 1.00 | 0.26 |
|  | Monobactam biosynthesis | 8 | 0.72 | 2 | 0.16 | 0.80 | 1.00 | 1.00 | 0.00 |
|  | Tyrosine metabolism | 16 | 1.45 | 3 | 0.17 | 0.77 | 1.00 | 1.00 | 0.11 |
|  | Glycine, serine and threonine metabolism | 33 | 2.98 | 5 | 0.17 | 0.77 | 1.00 | 1.00 | 0.42 |
|  | Ascorbate and aldarate metabolism | 18 | 1.63 | 3 | 0.22 | 0.66 | 1.00 | 1.00 | 0.00 |
|  | Arginine biosynthesis | 18 | 1.63 | 3 | 0.22 | 0.66 | 1.00 | 1.00 | 0.08 |
|  | beta-Alanine metabolism | 18 | 1.63 | 3 | 0.22 | 0.66 | 1.00 | 1.00 | 0.25 |
|  | Cutin, suberine and wax biosynthesis | 18 | 1.63 | 3 | 0.22 | 0.66 | 1.00 | 1.00 | 0.44 |
|  | Betalain biosynthesis | 3 | 0.27 | 1 | 0.25 | 0.61 | 1.00 | 1.00 | 0.00 |
|  | Phenylalanine metabolism | 11 | 0.99 | 2 | 0.26 | 0.58 | 1.00 | 1.00 | 0.47 |
|  | Cyanoamino acid metabolism | 29 | 2.62 | 4 | 0.26 | 0.58 | 1.00 | 1.00 | 0.00 |
|  | Linoleic acid metabolism | 4 | 0.36 | 1 | 0.32 | 0.50 | 1.00 | 1.00 | 1.00 |
|  | Valine, leucine and isoleucine biosynthesis | 22 | 1.99 | 3 | 0.32 | 0.50 | 1.00 | 1.00 | 0.15 |
|  | Pantothenate and CoA biosynthesis | 23 | 2.08 | 3 | 0.35 | 0.46 | 1.00 | 1.00 | 0.21 |
|  | Fatty acid biosynthesis | 56 | 5.06 | 6 | 0.39 | 0.40 | 1.00 | 1.00 | 0.01 |
|  | Histidine metabolism | 15 | 1.36 | 2 | 0.40 | 0.40 | 1.00 | 1.00 | 0.04 |
|  | Isoquinoline alkaloid biosynthesis | 6 | 0.54 | 1 | 0.43 | 0.36 | 1.00 | 1.00 | 0.50 |
|  | Butanoate metabolism | 17 | 1.54 | 2 | 0.46 | 0.33 | 1.00 | 1.00 | 0.14 |
|  | Tryptophan metabolism | 28 | 2.53 | 3 | 0.47 | 0.33 | 1.00 | 1.00 | 0.00 |
|  | Glyoxylate and dicarboxylate metabolism | 29 | 2.62 | 3 | 0.50 | 0.31 | 1.00 | 1.00 | 0.16 |
|  | Tropane, piperidine and pyridine alkaloid biosynthesis | 8 | 0.72 | 1 | 0.53 | 0.27 | 1.00 | 1.00 | 0.00 |
|  | Citrate cycle (TCA cycle) | 20 | 1.81 | 2 | 0.55 | 0.26 | 1.00 | 1.00 | 0.03 |
|  | Lysine biosynthesis | 9 | 0.81 | 1 | 0.57 | 0.24 | 1.00 | 1.00 | 0.00 |
|  | Carbon fixation in photosynthetic organisms | 21 | 1.90 | 2 | 0.58 | 0.24 | 1.00 | 1.00 | 0.04 |
|  | Thiamine metabolism | 22 | 1.99 | 2 | 0.61 | 0.22 | 1.00 | 1.00 | 0.00 |
|  | Phenylalanine, tyrosine and tryptophan biosynthesis | 22 | 1.99 | 2 | 0.61 | 0.22 | 1.00 | 1.00 | 0.15 |
|  | Pyruvate metabolism | 22 | 1.99 | 2 | 0.61 | 0.22 | 1.00 | 1.00 | 0.17 |
|  | Arginine and proline metabolism | 34 | 3.07 | 3 | 0.61 | 0.22 | 1.00 | 1.00 | 0.17 |
|  | Caffeine metabolism | 10 | 0.90 | 1 | 0.61 | 0.21 | 1.00 | 1.00 | 0.00 |
|  | Arachidonic acid metabolism | 12 | 1.08 | 1 | 0.68 | 0.17 | 1.00 | 1.00 | 0.00 |
|  | Glutathione metabolism | 26 | 2.35 | 2 | 0.70 | 0.16 | 1.00 | 1.00 | 0.13 |
|  | Nicotinate and nicotinamide metabolism | 13 | 1.17 | 1 | 0.71 | 0.15 | 1.00 | 1.00 | 0.00 |
|  | Galactose metabolism | 27 | 2.44 | 2 | 0.72 | 0.14 | 1.00 | 1.00 | 0.03 |
|  | Inositol phosphate metabolism | 28 | 2.53 | 2 | 0.74 | 0.13 | 1.00 | 1.00 | 0.10 |
|  | Sulfur metabolism | 15 | 1.36 | 1 | 0.76 | 0.12 | 1.00 | 1.00 | 0.00 |
|  | Pentose and glucuronate interconversions | 16 | 1.45 | 1 | 0.78 | 0.11 | 1.00 | 1.00 | 0.22 |
|  | Sphingolipid metabolism | 17 | 1.54 | 1 | 0.80 | 0.10 | 1.00 | 1.00 | 0.00 |
|  | Cysteine and methionine metabolism | 46 | 4.16 | 3 | 0.80 | 0.10 | 1.00 | 1.00 | 0.01 |
|  | Lysine degradation | 18 | 1.63 | 1 | 0.82 | 0.09 | 1.00 | 1.00 | 0.00 |
|  | Propanoate metabolism | 20 | 1.81 | 1 | 0.85 | 0.07 | 1.00 | 1.00 | 0.00 |
|  | Glycerolipid metabolism | 21 | 1.90 | 1 | 0.87 | 0.06 | 1.00 | 1.00 | 0.00 |
|  | Pyrimidine metabolism | 38 | 3.43 | 2 | 0.87 | 0.06 | 1.00 | 1.00 | 0.03 |
|  | Starch and sucrose metabolism | 22 | 1.99 | 1 | 0.88 | 0.06 | 1.00 | 1.00 | 0.14 |
|  | Fatty acid elongation | 23 | 2.08 | 1 | 0.89 | 0.05 | 1.00 | 1.00 | 0.00 |
|  | Phosphatidylinositol signaling system | 26 | 2.35 | 1 | 0.92 | 0.04 | 1.00 | 1.00 | 0.03 |
|  | Glycolysis / Gluconeogenesis | 26 | 2.35 | 1 | 0.92 | 0.04 | 1.00 | 1.00 | 0.12 |
|  | Folate biosynthesis | 27 | 2.44 | 1 | 0.92 | 0.03 | 1.00 | 1.00 | 0.00 |
|  | alpha-Linolenic acid metabolism | 28 | 2.53 | 1 | 0.93 | 0.03 | 1.00 | 1.00 | 0.00 |
|  | Diterpenoid biosynthesis | 28 | 2.53 | 1 | 0.93 | 0.03 | 1.00 | 1.00 | 0.01 |
|  | Phenylpropanoid biosynthesis | 46 | 4.16 | 2 | 0.93 | 0.03 | 1.00 | 1.00 | 0.04 |
|  | Terpenoid backbone biosynthesis | 30 | 2.71 | 1 | 0.94 | 0.03 | 1.00 | 1.00 | 0.00 |
|  | Fatty acid degradation | 37 | 3.34 | 1 | 0.97 | 0.01 | 1.00 | 1.00 | 0.00 |
|  | Valine, leucine and isoleucine degradation | 37 | 3.34 | 1 | 0.97 | 0.01 | 1.00 | 1.00 | 0.01 |
|  | Ubiquinone and other terpenoid-quinone biosynthesis | 38 | 3.43 | 1 | 0.97 | 0.01 | 1.00 | 1.00 | 0.00 |
|  | Purine metabolism | 63 | 5.69 | 2 | 0.98 | 0.01 | 1.00 | 1.00 | 0.00 |
|  | Glucosinolate biosynthesis | 65 | 5.87 | 2 | 0.99 | 0.01 | 1.00 | 1.00 | 0.00 |
|  | Porphyrin and chlorophyll metabolism | 48 | 4.34 | 1 | 0.99 | 0.00 | 1.00 | 1.00 | 0.03 |

All pathways shown in the table are potential target metabolic pathways with pathway impacts above 0.1; Total Cmpd, total number of compounds in the pathway; Hits, the number of actually matched compounds in the pathway; Holm adjust, p value adjusted by the Holm–Bonferroni method; FDR, p value adjusted using the False Discovery Rate; Impact, pathway impact value.
